# The CRL4^DTL^ E3 ligase induces degradation of the DNA replication initiation factor TICRR/TRESLIN specifically during S phase

**DOI:** 10.1093/nar/gkab805

**Published:** 2021-09-17

**Authors:** Kimberlie A Wittig, Courtney G Sansam, Tyler D Noble, Duane Goins, Christopher L Sansam

**Affiliations:** Department of Cell Biology, University of Oklahoma Health Sciences Center, Oklahoma City, OK 73104, USA; Cell Cycle and Cancer Biology Research Program, Oklahoma Medical Research Foundation, Oklahoma City, OK 73104, USA; Cell Cycle and Cancer Biology Research Program, Oklahoma Medical Research Foundation, Oklahoma City, OK 73104, USA; Department of Cell Biology, University of Oklahoma Health Sciences Center, Oklahoma City, OK 73104, USA; Cell Cycle and Cancer Biology Research Program, Oklahoma Medical Research Foundation, Oklahoma City, OK 73104, USA; Cell Cycle and Cancer Biology Research Program, Oklahoma Medical Research Foundation, Oklahoma City, OK 73104, USA; Department of Cell Biology, University of Oklahoma Health Sciences Center, Oklahoma City, OK 73104, USA; Cell Cycle and Cancer Biology Research Program, Oklahoma Medical Research Foundation, Oklahoma City, OK 73104, USA

## Abstract

A DNA replication program, which ensures that the genome is accurately and wholly replicated, is established during G1, before the onset of S phase. In G1, replication origins are licensed, and upon S phase entry, a subset of these will form active replisomes. Tight regulation of the number of active replisomes is crucial to prevent replication stress-induced DNA damage. TICRR/TRESLIN is essential for DNA replication initiation, and the level of TICRR and its phosphorylation determine the number of origins that initiate during S phase. However, the mechanisms regulating TICRR protein levels are unknown. Therefore, we set out to define the TICRR/TRESLIN protein dynamics throughout the cell cycle. Here, we show that TICRR levels are high during G1 and dramatically decrease as cells enter S phase and begin DNA replication. We show that degradation of TICRR occurs specifically during S phase and depends on ubiquitin ligases and proteasomal degradation. Using two targeted siRNA screens, we identify CRL4^DTL^ as a cullin complex necessary for TICRR degradation. We propose that this mechanism moderates the level of TICRR protein available for replication initiation, ensuring the proper number of active origins as cells progress through S phase.

## Graphical Abstract

**Graphical Abstract:**
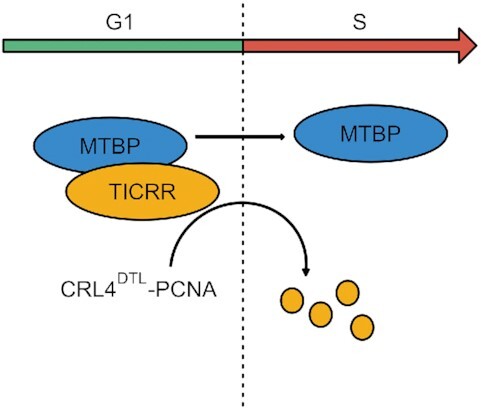
Two essential, limiting DNA replication factors are regulated at the G1/S transition through the destruction of TICRR/TRESLIN by the CRL4^DTL^ E3 ubiquitin ligase.

## INTRODUCTION

Deregulation of DNA replication fork initiation causes genome instability and promotes tumorigenesis (1–3). In higher eukaryotes, tens-of-thousands of replication forks initiate throughout S phase (4). Many oncogenes deregulate that process. They activate too many or too few DNA replication forks, and in turn, cause DNA damage (5). There is strong evidence that oncogenes cause aberrant replication initiation in human cancer, as markers of DNA damage from oncogene-induced hyper-replication are present at the earliest stages of tumorigenesis (1,2).

Replication initiation is regulated at two stages: replication origin licensing and origin firing. Licensing is the assembly of pre-replicative complexes (pre-RCs) onto genomic sites where DNA replication may later initiate. Origin licensing begins during late mitosis with the binding of origin recognition complex (ORC) proteins to potential replication origins (6,7). During G1, the CDC6 and CDT1 licensing factors recruit two mini-chromosome maintenance protein (MCM) heterohexamers to each potential origin (8). At the onset of S phase, replication licensing is blocked, and origin firing begins. Origin firing converts the MCMs in pre-RCs into active helicases and assembles two active bi-directional replication forks at each origin (7,8).

The inhibition of origin licensing during S phase ensures that all genomic segments are replicated only once during the cell cycle (9). Replication initiation itself plays a vital role in preventing the loading of pre-RCs during S phase, as the recruitment of PCNA to replication forks triggers the degradation of CDT1 (10–15). Degradation of CDT1 requires the CRL4^DTL^ E3 ubiquitin ligase complex, and PCNA serves as a platform on which the CRL4^DTL^ E3 ligase complex ubiquitinates CDT1 (10,11).

Although the number of licensed origins may set the upper limit of replication initiation, it does not generally determine DNA replication rates because pre-RCs are loaded onto more potential origins than are used during S phase (16–19). Instead, the replication initiation rate is controlled at the origin firing step and is limited by the availability of a set of origin firing factors (18–21). In yeast and *Xenopus laevis* embryos, for example, conserved origin firing factors are expressed at low levels relative to pre-RCs (20–22). Furthermore, overexpression of the same set of limiting origin firing factors in yeast and *X. laevis* embryos causes hyper-replication and DNA replication stress (20,21).

The limiting origin-firing factors in yeast, *X. laevis* embryos, and human cells are part of the mechanisms by which Cyclin-dependent and Dbf4-dependent kinases (CDKs and DDKs) trigger the recruitment and activation of initiation and replication factors to pre-RCs (23). In *Saccharomyces cerevisiae*, DDK phosphorylates MCMs, while CDK phosphorylates Sld2 and Sld3–Sld7, thereby promoting their interaction with Dpb11 (24–30). Phosphorylation of these factors is essential for converting the MCM complexes in pre-RCs into active helicases (25–30). Overexpression of Sld3, Sld2 and Dpb11, together with the DDK regulatory subunit Dbf4, is sufficient to increase the rate of replication fork firing in budding yeast (21). Similarly, the levels of Sld3, Sld2, Dpb11 and Dbf4 homologs in early *X. laevis* embryos are developmentally regulated, and they limit origin firing rates (20). The homologs of Sld3 and its binding partner Sld7 are called TICRR/TRESLIN and MTBP in higher eukaryotes (25,26,28,31,32). In human cells, the level of the CDK-phosphorylated form of TICRR/TRESLIN is limiting, as its overexpression can stimulate replication initiation and shorten S phase (33). Moreover, replication initiation factors are highly expressed in many cancers (34–40).

Although the levels of origin firing factors determine the rates of replication initiation, we know little about how the expression of these factors is regulated. Here, we inserted a tag into the endogenous locus of TICRR or MTBP in human HCT-116 colon cancer cells. By tagging endogenous TICRR and MTBP, we were able to measure changes in insoluble and total protein levels throughout the cell cycle. We show that insoluble and total expression levels of both TICRR and MTBP are cell cycle-regulated. Unexpectedly, TICRR levels are reduced as cells enter S phase. Utilizing proteasome and neddylation inhibitors followed by two targeted siRNA screens, we found that the degradation of TICRR in S phase is regulated via the CRL4^DTL^ E3 ubiquitin ligase complex, and this degradation is dependent on PCNA, CDC45, and DDK activity. This phase-specific degradation may be critical for regulating replication origin firing in a manner that prevents replication stress and subsequent genome instability.

## MATERIALS AND METHODS

### Cell culture

HCT-116 colon cancer cells (ATCC CCL-247) were cultured in McCoy's 5A medium (Corning) supplemented with 10% fetal bovine serum (FBS). For mitotic arrest, nocodazole (100ng/mL) was added to the media for 4 h. Cells were collected by manual shake-off and washed four times in phosphate-buffered saline (PBS) before re-seeding in culture medium. Time points were taken at intervals after release to monitor cell cycle progression. Flp-In T-rex 293 cells (Invitrogen, R78007) were cultured in Dulbecco's modified Eagle's medium (DMEM) (Corning) supplemented with 10% FBS. For transgene induction, doxycycline (2.5 ug/ml, Enzo) was added to the medium for 24 h. MG132 (20 uM, Selleck), MLN4924 (3 uM, Cayman Chemical), Palbociclib (100 nM, Selleck), T2AA (hydrochloride), (20 uM, Cayman Chemical), or NU-6102 (20 uM, Cayman Chemical) were added to the cell culture medium for the indicated times.

### Plasmid construction

For mClover knock-in cell lines (TICRR and MTBP), donor plasmids and CRISPR/Cas expression vectors were constructed. For the donor plasmids, synthetic DNA fragments (G blocks from Integrated DNA Technologies) with ∼200 bp homology arms were ligated using Gibson Cloning with cassettes containing the mAID-mClover coding sequence and neomycin or hygromycin selection cassettes from pMK289 or pMK290, respectively (pMK289 and pMK290 were gifts from Masato Kanemaki; Addgene plasmids #s 72827 and 72828; RRIDs:Addgene_72827 and Addgene_72828; Supplementary Tables S1 and S2) (41,42). For the CRISPR-Cas9 expression plasmids, oligos encoding gRNA sequences for the TICRR or MTBP C-termini were ligated into BbsI-digested pX330-U6-Chimeric_BB-CBh-hSpCas9, which was a gift from Feng Zhang (Addgene plasmid # 42230; http://n2t.net/addgene:42230; RRID:Addgene_42230; Supplementary Tables S1 and S2) (43). For the mfGFP-TICRR construct, overlapping DNA fragments encoding mfGFP and the human TICRR cDNA were cloned into pcDNA5/FRT/TO (Invitrogen) using isothermal assembly (25,42,44).

### Generation of stable cell lines

For mClover knock-in cell lines of TICRR and MTBP, HCT-116 cells were transfected (Mirus Bio) with pX330 and donor plasmids for TICRR or MTBP (41). After 48 h, the cells were split up into medium containing 100ug/mL (TICRR) or 150ug/mL (MTBP) hygromycin. After 14 days, selected cells were pooled and flow-sorted for positive mClover fluorescence (MoFloXDP11, Beckman-Coulter). Single-cell clones were isolated from the mClover positive cell populations and screened by flow cytometry for a shift in fluorescence and capillary electrophoresis for a shift in size compared to the parental HCT-116 cells.

For the stable 293 Flp-In T-rex TICRR cell line, we inserted mfGFP-TICRR(WT), mfGFP-TICRR(TESE), and mfGFP-TICRR(TASA) into pcDNA5/FRT/TO (Invitrogen) using isothermal assembly (33). 293 Flp-In T-rex cells were co-transfected with Flp recombinase (pOG44, Invitrogen) and the mfGFP-TICRR construct using TransIT-LT1 (Mirus Bio). Clones were selected with 200ug/mL hygromycin.

### siRNA transfections

siRNA was purchased from Sigma and Horizon Discovery (Supplementary Table S3). All siRNA was transfected with RNAiMax (Life Technologies) according to the manufacturer's instructions.

### Capillary electrophoresis

For nuclear lysate preparation, cells were harvested, washed in PBS, and resuspended in Buffer A (10 mM HEPES (pH 7.9), 10 mM KCl, 1.5 mM MgCl_2_, 0.34 M sucrose, 10% glycerol and 0.1% Triton) for 8 min on ice. Nuclei were centrifuged at 1300 × g and resuspended again in Buffer A containing benzonase. To prepare whole cell lysates, cells were harvested, washed and re-suspended in RIPA buffer (50 mM Tris (pH 8), 150 mM NaCl, 0.1% SDS, 1 mM EDTA, 0.5% DOC and 1% NP-40) for 30 min on ice. Nuclei were centrifuged at 12 000 RPM, and the supernatant was collected. Protein concentration was determined using the Bradford Assay (Bio-Rad). All capillary electrophoresis runs were normalized to Lamin (anti-Lamin, Cell Signaling 2032) or total protein (Protein Simple). Primary antibodies used include: anti-Treslin (Bethyl A303-472A), anti-MTBP (Santa Cruz, sc-137201), anti-GFP (Abcam, ab290), anti-CUL4B (Sigma, HPA011880), anti-CUL4A (Cell Signaling, 2699), anti-DDB1 (Santa Cruz Biotechnology, SC-137132), anti-CDT2 (Abcam, ab184548), anti-PCNA (Santa Cruz Biotechnology, PC10, sc-56), anti-CDC45L (Protein Tech 15678-1-AP), anti-WDR5 (Cell Signaling, 13105), anti-CDT1 (Abcam, ab202067), anti-p21 (Cell Signaling, 2947) and anti-SET8 (Cell Signaling, 2996). Images were generated with Compass Software (Protein Simple).

### Live cell flow cytometry

To harvest cells, media was aspirated, and cells were rinsed once in PBS. Cells were trypsinized, centrifuged, and resuspended in PBS. Samples were run on a FACSCelesta flow cytometer (BD Biosciences). Samples were analyzed using FlowJo (TreeStar). Cells were gated based on forward and side scatter area and represented as histograms.

### Fixed cell flow cytometry

To collect cells for flow cytometry analysis, the culture medium was collected, and cells were rinsed once with 1× PBS. For mitotic arrest experiments, the PBS rinse was also collected. Cells were then trypsinized, centrifuged at 500 × g for 3 min to pellet, and washed in 1× PBS. The samples were centrifuged, and the supernatant was aspirated. For analysis of insoluble protein samples, pellets were pre-permeabilized in cold CSK buffer (10 mM HEPES pH 7.4, 100 mM NaCl, 300 mM sucrose, 3 mM MgCl_2_, 0.5% Triton) on ice for 5 min to extract soluble proteins. After incubation, 1% bovine serum albumin (Sigma) in 1× PBS (PBS–BSA) was added, and samples were centrifuged at 2000 × g for 3 min. The supernatant was aspirated, and the pellets were fixed with 4% paraformaldehyde (PFA) for 12 min at 23°C. For total protein samples, the cells were fixed directly in 4% PFA for 12 min at 23°C. After incubation, PBS-BSA was added, and samples were centrifuged (2000 × g for insoluble protein, 500 × g for total protein). The supernatant was aspirated, and samples were blocked overnight in PBS + 2.5% normal goat serum (PBS–NGS) at 4°C. The next day, samples were centrifuged, and PBS-NGS was aspirated. Total protein samples were permeabilized in PBS + 0.5% Triton-X for 20 min at 23°C. Next, samples were incubated in primary antibody in dilution buffer (1× PBS, 2.5% NGS, 0.1% NP-40) for 1 h at 23°C. Additional dilution buffer was added, mixed, and the samples were centrifuged. The supernatant was aspirated, and the samples were incubated in secondary antibody in dilution buffer for 1 h at 23°C. Additional dilution buffer was added, mixed and the samples were centrifuged. The supernatant was aspirated, and the pellets were resuspended in PBS containing 20ug/ml RNase A and 50 ug/ml propidium iodide. EdU labeling experiments were done as described previously (33).

Samples were run on a FACSCelesta flow cytometer (BD Biosciences). Samples were analyzed using FlowJo (TreeStar). Single cells were gated based on area (PI-A) versus width (PI-W) (Supplementary Figure S2A). Positive fluorescence signal gating was based on a negative control. Negative controls were run for every experiment. For the TICRR-mClover and MTBP-mClover knock-in cell line experiments, the negative control was GFP antibody plus secondary antibody in the parental HCT-116 cell line (Supplementary Figure S2B). For quantification, samples were background subtracted and then normalized. To background subtract, the median signal intensity of the negative control sample was subtracted from the target sample's median intensity within the same gate. To normalize samples between two groups, the treatment sample was normalized to the control sample. The following antibodies were used: Anti-GFP (Abcam 290), Anti-GFP (Rockland 600-401-215S), Anti-MCM7 (Santa Cruz Biotechnology 47DC141), Alexa-488 goat anti-rabbit (Life Technologies) and Alexa-647 goat anti-mouse (Life Technologies).

The data is represented as bivariate pseudocolor dot plots. The colors within the pseudocolor plots represent the relative population density of cell populations over the plot area. Blue and green correspond to areas of lower cell density, red and orange are areas of high cell density, and yellow is mid-range.

### Statistics

Statistical analyses of single comparisons were performed using a two-tailed paired Student's *t*-test. Statistical analyses of multiple comparisons were performed using a two-way ANOVA with Dunnett correction. Data are reported as mean ± SD (unless otherwise noted) using GraphPad Prism version 8.1.2 (GraphPad Software). The analyzed number of samples is indicated in the figure legends. Asterisks indicate significance values as follows: ^∗^*P* < 0.05, ^∗∗^*P* < 0.01, ^∗∗∗^*P* < 0.001 and ^****^P < 0.0001.

## RESULTS

### TICRR protein levels are cell cycle regulated

**Figure 1.**
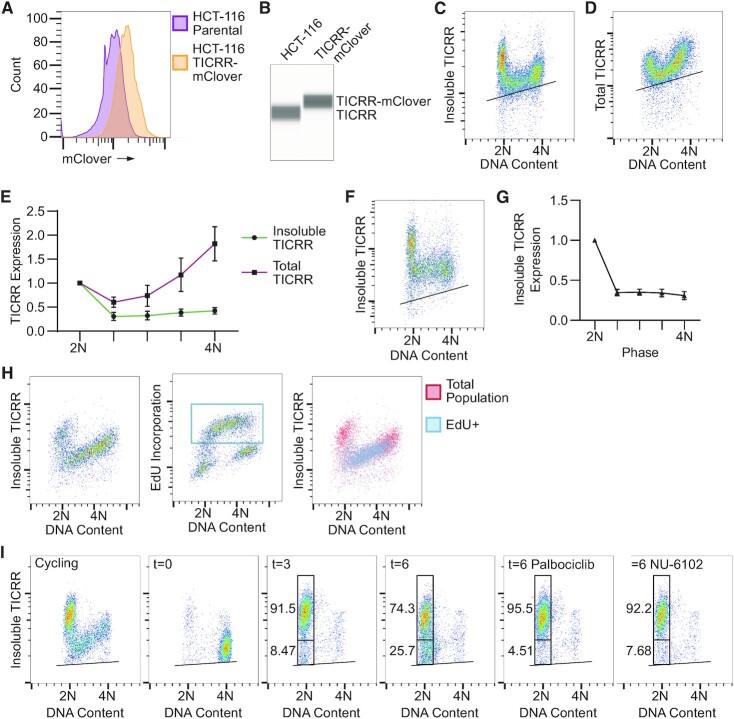
TICRR protein expression levels are cell cycle regulated. (**A**) Counts of live intact HCT-116 or HCT-116 TICRR-mClover cells with indicated mClover fluorescence intensities measured by flow cytometry. (**B**) Capillary electrophoresis of nuclear protein lysate from HCT-116 untagged and HCT-116 TICRR-mClover cells using an anti-TICRR antibody. Immuno-flow cytometry measurement of DNA content (propidium iodide) and (**C**) insoluble TICRR-mClover (anti-GFP, Ab290) or (**D**) total TICRR-mClover (anti-GFP) in TICRR-mClover tagged cells. (**E**) Quantification of median TICRR-mClover levels (background subtracted and normalized to 2N) for all cells over the indicated DNA content ranges from C and D. Means ± SD for 6 independent experiments are plotted. (**F**) Fixed cell flow cytometry measurement of DNA content (propidium iodide) and insoluble wild-type mfGFP-TICRR (anti-GFP) in 293 Flp-In T-Rex cells after 24-h doxycycline induction of the mfGFP-TICRR transgene. (**G**) Median mfGFP-TICRR levels from F quantified as in Figure 1E (*n* = 3 independent experiments). (**H**) Immuno-flow cytometry measurement of DNA content (propidium iodide) and (left) insoluble TICRR-mClover (anti-GFP, Alexa-488) or (middle) EdU incorporation (Alexa-647) in HCT-116 TICRR-mClover tagged cells. Box represents EdU + cells. Right dot plot shows EdU + cells (boxed population) overlayed on the insoluble TICRR dot plot. (**I**) Insoluble TICRR-mClover (anti-GFP) and DNA content at timepoints following release from mitotic arrest and treatment with Palbociclib (CDK4/6 inhibitor) or NU-6102 (CDK1/2 inhibitor). Values represent percent of cells within the 2N cell gate as demonstrated by the boxes. Black lines within bivariate density plots in C, D, F and I mark the upper limits of background signals from negative controls (see Supplementary Figure S2B–D).

We aimed to evaluate the dynamics of both insoluble and total levels of TICRR throughout the cell cycle in mammalian cells. Previous reports have established a flow cytometry assay to detect changes in protein-chromatin association throughout the cell cycle (45–48). We initially tried this approach with a commercially available TICRR antibody against the endogenous protein, but it provided no signal over background (data not shown). Therefore, we tagged the C-terminus of TICRR with mClover at the endogenous locus in HCT-116 cells such that all of the expressed protein was tagged (Figure 1A, B and Supplementary Figure S1). The proliferation rate of this tagged cell line was the same as the parental HCT-116 untagged cell line (Supplementary Figure S1C). Using this TICRR-mClover-tagged cell line, we were able to detect TICRR using an anti-GFP antibody. We used flow cytometry to assess the amount of TICRR in the ‘detergent-resistant’ fraction of the nucleus using CSK buffer to extract all except chromatin- or nuclear matrix-associated proteins (Figure 1C). We refer to the CSK-resistant pool as ‘insoluble.’ We tested the CSK extraction protocol on MCM7. CSK extraction removed all soluble MCM7 from G2/M (4N) cells but not the chromatin-bound MCM7 in G1 and S phase cells (Supplementary Figure S2E). Flow cytometry measurement of insoluble TICRR and DNA content revealed TICRR was unexpectedly highest in G1 (2N) cells and sharply decreased with S phase entry (Figure 1C, E, and Supplementary Figure S2).

To determine whether the changes in insoluble, nuclear TICRR were due to overall changes in protein levels, we used flow cytometry to measure the total levels of TICRR in the mClover knock-in cells. We found that the expression of TICRR was highest in G2/M (4N) cells, decreased in G1 (2N) cells with cell division, and was lowest in early S phase (Figure 1D-E and Supplementary Figure S2). This is consistent with a previous report of endogenous TICRR expression in U2OS cells by immunoblotting (49). Importantly, although the overall level of TICRR decreased between G2/M and G1 phases, the level of insoluble TICRR increased, demonstrating that the accumulation of insoluble, nuclear TICRR in G1 is not due to changes in its expression. In contrast, both total and insoluble TICRR decreased with S phase entry, suggesting unloading of TICRR from chromatin or the nuclear matrix during early S phase is at least partially due to the degradation of TICRR protein.

We wanted to confirm the decrease in bound TICRR levels in an unrelated cell line. To do this, we introduced a doxycycline-inducible transgene encoding an N-terminal mfGFP-tagged wild-type TICRR into a single genomic locus in 293 Flp-In T-rex cells (44). After inducing expression for 24 h, insoluble mfGFP-TICRR levels were highest in G1 (2N) cells and decreased with S phase entry (Figure 1F, G). The identical cell cycle expression pattern of TICRR in HCT-116 and 293 Flp-In T-rex cells demonstrates that the decrease in TICRR levels at G1/S is through a post-transcriptional mechanism and suggests that the decrease in bound TICRR levels at the G1/S transition is a general regulatory mechanism in human cells.

### Decrease of TICRR protein levels requires entry into S phase

Next, we tested whether TICRR downregulation during S phase was correlated with DNA synthesis. To do this, we pulse-labeled asynchronously cycling HCT-116 TICRR-mClover cells for 15 min with a thymidine analog, EdU, and quantified EdU, TICRR and DNA content in the same cells using flow cytometry (Figure 1H). These results indicated that all cells with reduced TICRR levels were EdU-positive and vice versa (Figure 1H). This confirmed that the decrease in TICRR levels that occurred at the G1/S transition coincided with DNA synthesis.

Following this result, we questioned whether TICRR degradation required G1 exit via the activities of G1 or S phase CDKs. To control the timing of CDK inhibition, we synchronized the HCT-116 TICRR-mClover cells with nocodazole. TICRR-mClover cells released from nocodazole-induced mitotic arrest proceeded into G1 within 3 h and began entering S phase within 6 h (Figure 1I). As expected, insoluble TICRR levels were low in nocodazole-arrested cells (t = 0 hrs.), accumulated in G1 cells (*t* = 3 h), and then dropped as cells entered S phase (*t* = 6 h; Figure 1I). Treatment with Palbociclib, a CDK4/6 inhibitor, added immediately upon release from the nocodazole arrest, halted S phase entry and prevented the decrease in TICRR levels at 6 h (Figure 1I) (50). Notably, inhibition of CDK4/6 activity did not impair the accumulation of insoluble TICRR during G1. Treatment of cells with NU-6102, a selective CDK1/2 inhibitor, added 3 h after release, also inhibited S phase entry and prevented the decrease in TICRR levels at 6 h (Figure 1I) (51). NU-6102 was not added immediately like Palbociclib because this compound inhibits cytokinesis (52). Together, these data suggest that S-phase entry is required for the decrease in TICRR protein levels.

### Cullin E3 ubiquitin ligases degrade TICRR during S phase

**Figure 2.**
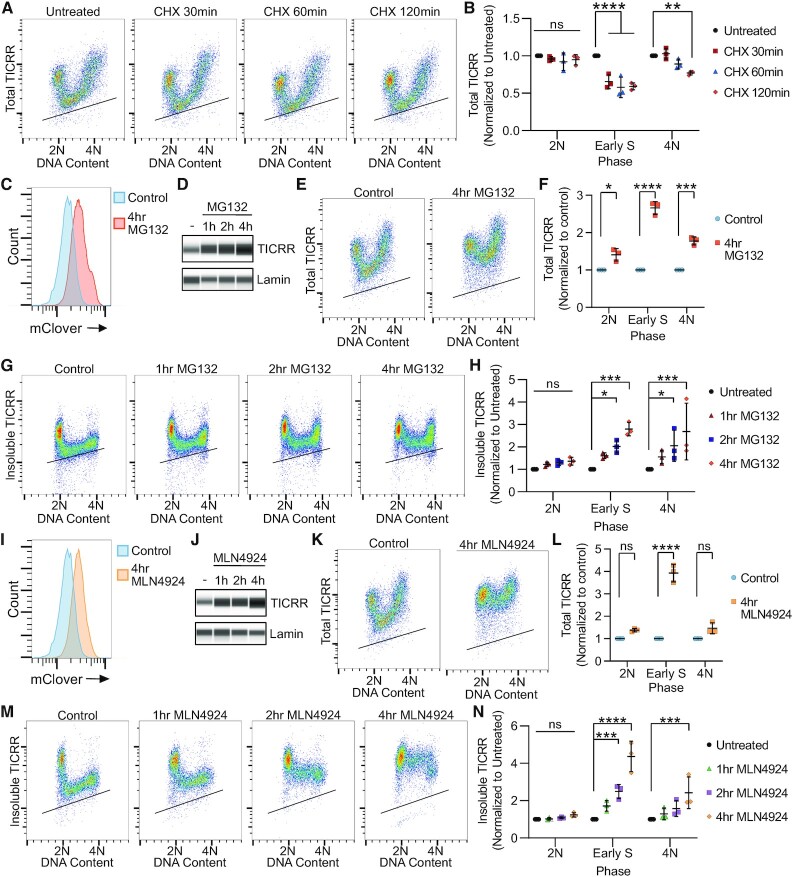
Cullin E3 ubiquitin ligases degrade TICRR during S phase. (**A**) Immuno-flow cytometry measurement of total TICRR-mClover (anti-GFP) and DNA content (propidium iodide) after cycloheximide (CHX) treatment. (**B**) Quantification of median TICRR-mClover levels of cells in A (*n* = 3 independent experiments). (**C**) Counts of live untreated (Control) or MG132-treated (20uM) HCT-116 TICRR-mClover tagged cells with indicated mClover fluorescence intensities measured by flow cytometry. (**D**) Capillary electrophoresis of nuclear protein lysates after treatment with MG132 (20uM) after 1, 2 and 4 h and probed for TICRR-mClover (anti-GFP) and Lamin (anti-Lamin). (**E**) Immuno-flow cytometry measurement of total TICRR-mClover (anti-GFP) and DNA content (propidium iodide) after MG132 (20uM) treatment. (**F**) Quantification of median TICRR-mClover levels of cells in E (*n* = 3 independe(A)nt experiments). (**G**) Immuno-flow cytometry measurement of insoluble TICRR-mClover (anti-GFP) and DNA content (propidium iodide) after MG132 (20 uM) treatment. (**H**) Quantification of median TICRR-mClover levels of cells in G (*n* = 3 independent experiments). (**I**) Counts of live untreated (Control) or MLN4924-treated (3uM) HCT-116 TICRR-mClover tagged cells with indicated mClover fluorescence intensities measured by flow cytometry. (**J**) Capillary electrophoresis of nuclear protein lysates after treatment with MLN4924 (3 uM) after 1, 2 and 4 h and probed for TICRR (anti-GFP) and Lamin (anti-Lamin). (**K**) Immuno-flow cytometry measurement of total TICRR-mClover (anti-GFP) and DNA content (propidium iodide) after MLN4924 (3 uM) treatment. (**L**) Quantification of median TICRR-mClover levels of cells in K (*n* = 3 independent experiments). (**M**) Immuno-flow cytometry measurement of insoluble TICRR-mClover (anti-GFP) and DNA content (propidium iodide) after MLN4924 (3 uM) treatment. (**N**) Quantification of median TICRR-mClover levels of cells in M (*n* = 3 independent experiments). Black lines within bivariate density plots in A, E, G, K and M mark the upper limits of background signals from negative controls. Midlines and error bars in scatterplots in B, F, H, L and N mark the mean ± SD. *P*-values in B, F, H, L and N are from two-way ANOVA with Dunnett's Post Hoc Test (^∗^*P*< 0.05, ^∗∗^*P*< 0.01, ^∗∗∗^*P*< 0.001 and ^****^P< 0.0001).

We next sought to evaluate if TICRR levels decrease at the G1/S transition due to protein degradation. To test this, we first used immuno-flow cytometry to measure TICRR-mClover levels in G1, S and G2/M after inhibiting translation with cycloheximide. S phase TICRR levels decreased 40% after 30 min and 50% after 1 and 2 h, with no effect on G1 TICRR levels (Figure 2A, B). TICRR expression in G2/M cells slightly decreased with cycloheximide treatment over time, but the effect was delayed compared to S phase (Figure 2A, B). Next, we treated asynchronously cycling cells with the proteasome inhibitor MG132. Four-hour-MG132-treatment increased TICRR-mClover fluorescence in live cells (Figure 2C). Furthermore, TICRR levels, as measured by capillary electrophoresis, were increased in nuclear lysates of MG132-treated cells (Figure 2D). These results confirm previously published data showing that the proteasome regulates TICRR levels (49). To determine when during the cell cycle TICRR is degraded by the proteasome, we treated asynchronously growing cells with MG132 and then measured DNA content and total or insoluble TICRR by flow cytometry (Figure 2E–H). MG132 treatment increased TICRR levels in all phases of the cell cycle but had the strongest effect during S phase, indicating that the rate of proteasomal degradation of TICRR is highest in cells undergoing DNA replication (Figure 2E–H).

We next determined whether TICRR degradation at the G1/S transition is via a cullin E3 ubiquitin ligase pathway, as had been previously proposed (49). To test this, we utilized the neddylation activating enzyme (NAE) inhibitor, MLN4924, as all cullin proteins are activated through the addition of NEDD8, a ubiquitin-like molecule, to the cullin subunit (53,54). After 4 h of MLN4924 treatment, we detected an increase in TICRR-mClover fluorescence, which we confirmed by capillary electrophoresis of nuclear protein lysates (Figure 2I, J). Analysis of total TICRR protein by flow cytometry after MLN4924 treatment demonstrated the increase in TICRR levels occurs specifically during S phase (Figure 2K, L), and the changes in insoluble TICRR levels mirrored the changes in total TICRR levels (Figure 2M, N). Collectively, these data demonstrate the decrease in both insoluble and total TICRR levels is due to TICRR protein degradation via a cullin-dependent pathway during S phase.

### TICRR degradation in S phase requires CUL4, DDB1 and DTL/CDT2

**Figure 3.**
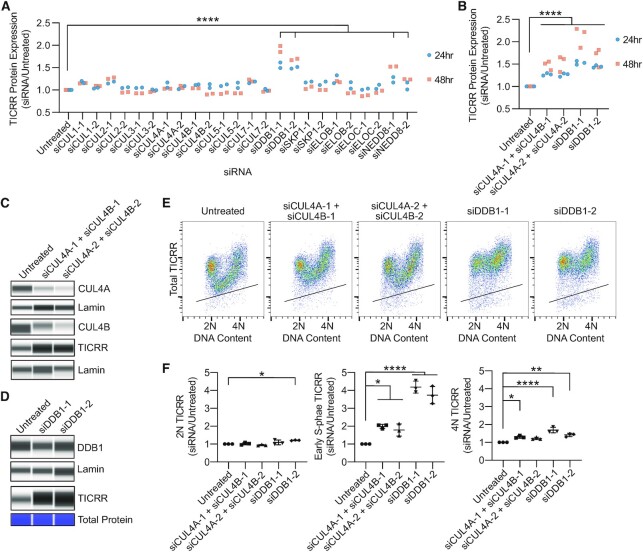
CUL4 and DDB1 knockdown increase TICRR protein levels in S phase. (**A, B**) HCT-116 TICRR-mClover cells were transfected with indicated siRNA, and TICRR-mClover signal was measured after 24 or 48 h by live cell flow cytometry. Median mClover signals for at least 10 000 live, intact cells were normalized to median signal from untreated cells. (**C**) Capillary electrophoresis of whole cell lysates 24 h after siRNA transfection probed for CUL4A (anti-CUL4A), CUL4B (anti-CUL4B), TICRR (anti-GFP) and Lamin (anti-Lamin). (**D**) Capillary electrophoresis of whole cell lysates 24 h after siRNA knockdown of DDB1 probed for DDB1 (anti-DDB1) and TICRR (anti-GFP) normalized to Lamin (anti-Lamin) or total protein. (**E**) Immuno-flow cytometry measurement of total TICRR-mClover (anti-GFP) and DNA content (propidium iodide) 24 h after siRNA transfection. Black line represents upper limit of background signal from negative control. (**F**) Quantification of median TICRR-mClover levels in 2N, early-S, and 4N cells from E (*n* = 3 independent experiments). Values are normalized to untreated cells. Midlines and error bars mark the mean ± SD. All *P*-values are from two-way ANOVA with Dunnett's Post Hoc Test (^∗^*P*< 0.05, ^∗∗^*P*< 0.01 and ^****^P< 0.0001).

To identify the specific cullin E3 ubiquitin ligase that induces degradation of TICRR at the G1/S transition, we performed a targeted siRNA screen to reduce expression of NEDD8 and every known cullin and cullin adapter protein to evaluate the effect on TICRR protein levels (Supplementary Table S3). We used live-cell flow cytometry to assess TICRR protein levels 24 and 48 h after siRNA transfection (Figure 3A). Consistent with the effect of MLN4924 treatment, siRNA knockdown of NEDD8 resulted in a significant increase in TICRR levels (Figures 2I–N and 3A). TICRR-mClover expression, as measured by flow cytometry (Figure 3A, B) or capillary electrophoresis (Figure 3D), was also significantly increased after DDB1 knockdown. DDB1 is the adaptor protein for CUL4A and CUL4B E3 complexes, but we did not detect an increase in TICRR levels after single knockdown of CUL4A or CUL4B (Figure 3A) (55). As CUL4A and CUL4B can act redundantly, we performed a double knockdown of CUL4A and CUL4B in the same cells (56). This resulted in a significant increase in TICRR protein levels (Figure 3B, C). To more carefully determine how CUL4A + B or DDB1 knockdown affected TICRR, we measured total TICRR protein levels and DNA content after 24 h using immuno-flow cytometry. This resulted in significant TICRR protein accumulation in S phase with minimal effects on G1 (2N) or G2/M (4N) cells (Figure 3E, F). Although knockdown of CUL4A + B or DDB1 is known to cause re-replication, we observed very little change in the percentage of cells in S phase or in the rate of EdU incorporation 24 h after CUL4A + B or DDB1 siRNA knockdown (Supplementary Figure S3). Altogether, our results demonstrate that degradation of TICRR at the G1/S transition requires the CUL4-DDB1 E3 ubiquitin ligase.

**Figure 4.**
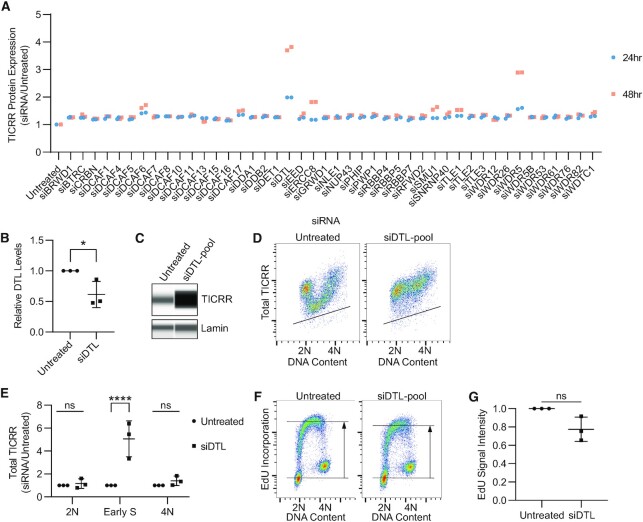
DTL knockdown increases TICRR protein levels in S phase. (**A**) TICRR-mClover signal, measured as in Figure 3A, 24 or 48 h after siRNA transfection. (**B**) Quantification of capillary electrophoresis of whole cell lysates prepared 24 h after siDTL-pool transfection probed for DTL/CDT2 (anti-CDT2) normalized to Lamin (anti-Lamin) (*n* = 3). *P*-value is from Student's *t*-test (∗*P*< 0.05) (**C**) Capillary electrophoresis of whole cell lysates 24 h after siDTL-pool transfection probed for TICRR (anti-GFP) and Lamin (anti-Lamin). (**D**) Immuno-flow cytometry measurement of total TICRR-mClover (anti-GFP) and DNA content (propidium iodide) 24 h after siDTL-pool transfection. Black line represents upper limit of background signal from negative control. (**E**) Quantification of median TICRR-mClover signal in 2N, early-S, and 4N cells from D (*n* = 3 independent experiments). Values are normalized to untreated cells. Midlines and error bars mark the mean ± SD. *P*-values are from two-way ANOVA with Dunnett's Post Hoc Test (^∗^*P*< 0.05). (**F**) EdU incorporation and DNA content (propidium iodide) were measured by flow cytometry 24 h after siDTL-pool transfection. Gray box represents EdU+ cells. Horizonal lines represent median intensity of G1 (bottom) and EdU+ (top) cell populations. (**G**) Median EdU signal intensity was normalized to that of untreated cells. *P*-value is from Student's *t*-test, ns.

All known ubiquitin E3 ligases have specificity factors that limit their activity to cognate substrates. We next wanted to identify the substrate receptor providing specificity for TICRR degradation. To do this, we performed a targeted siRNA screen against all 44 known nuclear DCAFs (DDB1 and CUL4-associated factors) using siRNA pools consisting of four siRNAs against each target (Supplementary Table S3) (57). Cells were collected 24 or 48 h after siRNA transfection and analyzed via live-cell flow cytometry (Figure 4A). Through this screen, we identified two candidate DCAFs that resulted in increased TICRR levels: DTL/CDT2 and WDR5 (Figure 4A).

Total TICRR levels were significantly increased 24 h after DTL siRNA knockdown (Figure 4B–E and Supplementary Figure S4). Each of three DTL siRNAs effectively reduced DTL and increased TICRR expression, indicating that the effect was not caused by off-target knockdown. Measuring TICRR 24 h after DTL knockdown using immuno-flow cytometry demonstrates the increase in TICRR protein levels is specific to S phase with minimal effects on 2N or 4N cells (Figure 4D-E). In contrast, 24 h after WDR5 knockdown by an siRNA pool, S phase TICRR levels were bi-modal (Supplementary Figure S5A–C). Some cells were able to decrease TICRR levels to that of untreated cells, but other cells maintained high TICRR levels comparable to DTL knockdown (Supplementary Figure S5A–C). Only one of the four siRNAs in the WDR5 pool increased TICRR expression on its own (Supplementary Figure S5D, E), even though each of the four siRNAs knocked down WDR5. Therefore, we conclude that DTL is a DCAF required for TICRR destruction.

We next evaluated DNA replication by performing EdU flow cytometry. Cells were pulse-labeled with EdU 24 h after siRNA transfection for 15 min. We did not observe an accumulation of >4N DNA, suggesting that there is not a strong re-replication phenotype after 24 h, but we saw an increase in late S phase cells that were not incorporating EdU. This suggests that DTL knockdown results in a small fraction of cells halting DNA synthesis before S phase is complete (Figure 4D-F). Importantly, the effect of DTL knockdown on TICRR expression occurs throughout S phase, so the increase in TICRR protein cannot be attributed to a decrease in DNA synthesis in late S phase.

The CUL4-DDB1-DTL/CDT2 (CRL4^DTL^) complex ubiquitinates other replication factors at the G1/S transition. Therefore, we hypothesized that CRL4^DTL^ triggers TICRR degradation indirectly through the ubiquitination of one of its known substrates (58). We focused our experiments on three substrates known to be ubiquitinated by CRL4^DTL^ during S phase: CDT1, p21 and SET8. If DTL knockdown causes TICRR overexpression by dysregulating another protein, loss of that protein should mask the effect. Therefore, we measured TICRR protein levels in cells transfected with siRNAs against CDT1, p21 or SET8 with or without siDTL (Supplementary Table S3). As expected, DTL knockdown caused CDT1, p21, and SET8 overexpression (Supplementary Figure S6A). Transfection of CDT1, p21, or SET8 siRNA prevented the overexpression of the respective target upon DTL knockdown but did not affect TICRR levels (Supplementary Figure S6B-D). These data demonstrate that CRL4^DTL^ does not indirectly trigger the degradation of S phase TICRR through the destruction of CDT1, p21 or SET8.

### TICRR degradation in S phase requires PCNA

**Figure 5.**
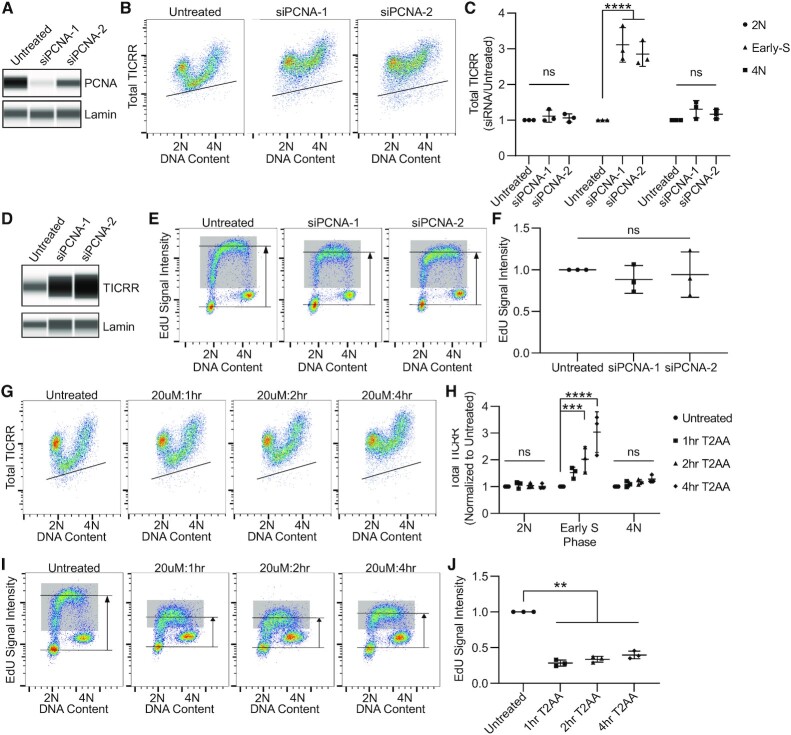
S phase TICRR degradation requires PCNA. (**A**) Capillary electrophoresis of whole cell lysates 24 h after siPCNA transfection measuring PCNA (anti-PCNA) and Lamin A/C (anti-lamin A/C). (**B**) Immuno-flow cytometry of DNA content (propidium iodide) and total TICRR-mClover (anti-GFP) 24 h after siPCNA transfection. (**C**) Quantification of median mClover signal in 2N, early-S, and 4N cells from B (*n* = 3 independent experiments). Values are normalized to untreated cells. (**D**) Capillary electrophoresis of whole cell lysates 24 h after siPCNA transfection measuring TICRR (anti-GFP) and Lamin A/C (anti-Lamin A/C). (E-F) EdU flow cytometry 24 h after siPCNA transfection. Gray box represents EdU+ cells. Horizonal lines represent median intensity of G1 (bottom) and EdU+ (top) cell populations. Median EdU signal intensity was normalized to that of untreated cells. (**G**) Immuno-flow cytometry of DNA content (propidium iodide) and total TICRR-mClover (anti-GFP) after T2AA (20 uM) treatment. (**H**) Quantification of median mClover signal in 2N, early-S, and 4N cells from G (*n* = 3 independent experiments). (**I, J**) EdU flow cytometry after 1, 2 or 4 h of T2AA (20uM) treatment. Gray box represents EdU+ cells. Horizonal lines represent median intensity of G1 (bottom) and EdU+ (top) cell populations. Median EdU signal intensity was normalized to that of untreated cells. Black lines within bivariate density plots in B and G mark the upper limits of background signals from negative controls. Midlines and error bars in scatterplots in C, F, H and J mark the mean ± SD. *P*-values are from two-way ANOVA with Dunnett's Post Hoc Test (^∗∗^*P*< 0.01, ^∗∗∗^*P*< 0.001 and ^****^P< 0.0001).

Although the degradation of most known CRL4^DTL^ substrates depends on their association with PCNA through a conserved PCNA-Interacting Protein motif (PIP box) degron sequence, CRL4^DTL^ ubiquitination of CHK1 or GCN5 does not require PCNA (59–61). TICRR lacks an obvious PIP degron within its sequence. Therefore, we aimed to evaluate the necessity of PCNA for TICRR degradation. Total TICRR levels increased specifically in S phase following siRNA knockdown of PCNA after 24 h (Figure 5A–D). Pulse labeling these cells with EdU demonstrates DNA replication is proceeding relatively normally 24 h after knockdown (Figure 5E, F). This suggests the majority of PCNA in these cells is involved in targeted protein degradation. Next, we used the competitive PIP box chemical inhibitor T2AA to prevent the interaction between PCNA and PIP-box containing proteins (62). Treatment with T2AA results in a time-dependent increase in total TICRR protein levels during S phase (Figure 5G-H). Pulse-labeling these T2AA treated cells with EdU demonstrates a significant reduction in DNA synthesis (Figure 5I, J). Although PCNA siRNA and T2AA have differing effects on DNA replication, both treatments increased levels of TICRR during S phase, demonstrating that PCNA is required for TICRR protein degradation.

### MTBP protein levels do not decrease in S phase

**Figure 6.**
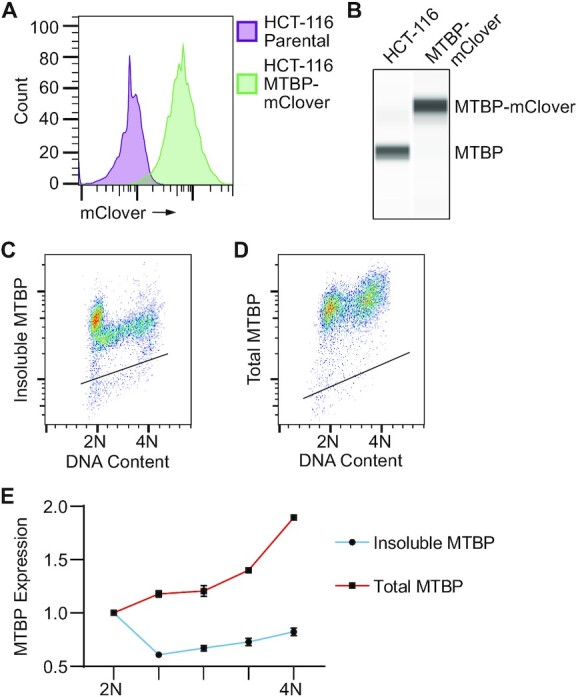
MTBP protein levels do not decrease in S phase. (**A**) Counts of live intact HCT-116 parental or HCT-116 MTBP-mClover tagged cells with indicated mClover fluorescence intensities measured by flow cytometry. (**B**) Capillary electrophoresis of nuclear protein lysate from HCT-116 parental and HCT-116 MTBP-mClover cells using an antibody against endogenous MTBP. Fixed cell flow cytometry measurement of DNA content (propidium iodide) and (**C**) insoluble MTBP-mClover (anti-GFP) or (**D**) total MTBP-mClover (anti-GFP). (**E**) Quantification of MTBP levels (background subtracted and normalized to 2N) from C and D over the indicated DNA content ranges (*n* = 3 independent experiments). Data represented as mean ± SD. Black lines within bivariate density plots in C and D represent upper limits of background signals from negative control.

TICRR and MTBP function together for DNA replication initiation and form a complex throughout the cell cycle, so we reasoned that their overall expression or chromatin association might be co-regulated (31,63). Therefore, we also tested whether MTBP levels changed across the cell cycle like those of TICRR. To do this, we tagged the C-terminus with mClover at the endogenous locus of MTBP in HCT-116 cells (Supplementary Figure S1). We isolated a knock-in clone with homogeneous expression of mClover in which all MTBP protein was tagged (Figure 6A, B). The proliferation rate of this clone was the same as the parental HCT-116 untagged cell line (Supplementary Figure S1C). Using an anti-GFP antibody in these MTBP-mClover cells, we were able to detect the levels of both insoluble and total MTBP throughout the cell cycle using flow cytometry (Figure 6C, D and Supplementary Figure S2). Like TICRR, insoluble MTBP was at its highest levels during G1 and decreased when cells entered S phase (Figure 6C and E). Notably, the degree of change in insoluble MTBP at G1/S was less than TICRR (Figure 6C and E and Figure 1C and E). Although total MTBP levels were highest in G2/M (4N) cells and decreased with cell division in G1 (2N) cells, total MTBP levels did not decrease at the G1/S transition as seen with TICRR (Figure 6D, E).

### CRL4^DTL^ is required for MTBP release from the insoluble nuclear fraction

**Figure 7.**
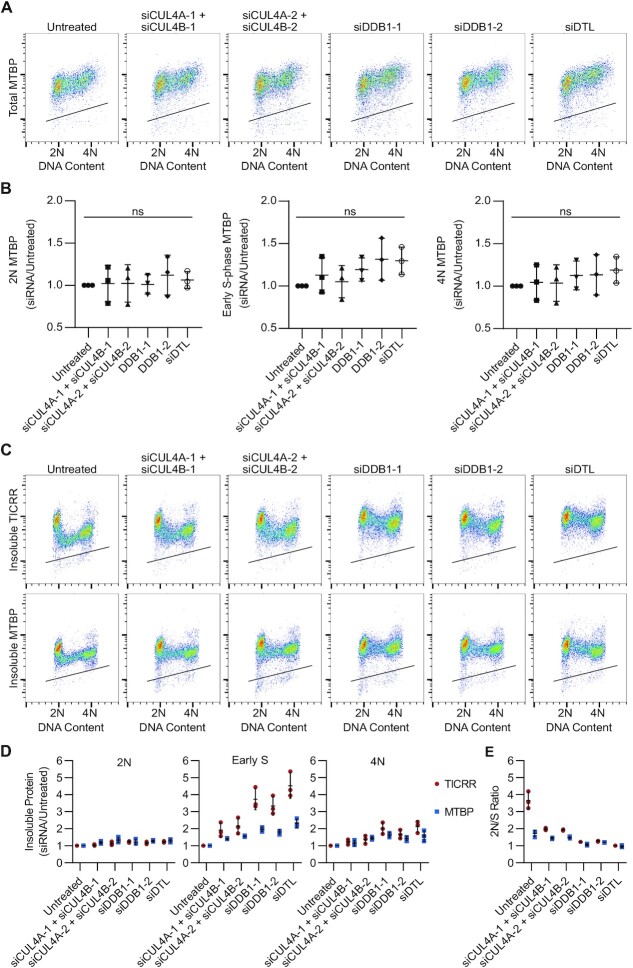
CRL4^DTL^ is required for MTBP release from the insoluble nuclear fraction. (**A**) Immuno-flow cytometry measurement of total MTBP (anti-GFP) 24 h after siRNA transfection. (**B**) Quantification of median MTBP-mClover signal in 2N, early-S, and 4N cells from A (*n* = 3 independent experiments). Values are normalized to untreated cells. *P*-values are from two-way ANOVA with Dunnett's Post Hoc Test (ns *P*≥ 0.05). (**C**) Immuno-flow cytometry measurement of insoluble TICRR-mClover (anti-GFP) and MTBP-mClover (anti-GFP) 24 h after siRNA transection. (**D**) Quantification of median TICRR-mClover and MTBP-mClover signals in 2N, early-S, and 4N cells from C (*n* = 3 independent experiments). Statistics shown in Supplementary Table S4. Values are normalized to untreated cells. (**E**) 2N/S ratios of TICRR-mClover and MTBP-mClover cells from C, 24 h after siRNA transfection (*n* = 3 independent experiments). Black lines within bivariate density plots in A and C represent upper limits of background signals from negative control. Midlines and error bars in scatterplots in B, D, and E mark the mean ± SD. *P*-values are from two-way ANOVA with Dunnett's Post Hoc Test (^∗∗^*P*< 0.01, ^∗∗∗^*P*< 0.001 and ^****^P< 0.0001).

To determine if MTBP expression is also controlled by CRL4^DTL^, we transfected the HCT-116 MTBP-mClover cells with siRNA against CUL4A + B, DDB1, or DTL and measured MTBP levels using flow cytometry (Figure 7). Unlike TICRR expression, total MTBP protein expression was not significantly increased in any cell cycle phase after siRNA knockdown (Figure 7A, B). Therefore, we conclude that MTBP is not a target of CRL4^DTL^, and CRL4^DTL^ does not affect TICRR protein levels indirectly through MTBP.

In contrast to its total protein levels, insoluble MTBP levels were increased by CRL4^DTL^ knockdown (Figure 7C, D). Like TICRR, insoluble MTBP was increased most in S phase and least in G1 after CRL4^DTL^ knockdown (Figure 7C, D and Supplementary Table S4). Although insoluble TICRR levels were affected more than MTBP, the drop in insoluble protein levels that normally occurs at the G1/S transition for both proteins was nearly completely inhibited by DDB1 or DTL knockdown (Figure 7E). These data indicate that although CRL4^DTL^ does not regulate MTBP expression, it does reduce the association of the TICRR–MTBP complex with chromatin or the nuclear matrix during S phase, possibly through the degradation of TICRR.

### TICRR degradation requires CDC45

**Figure 8.**
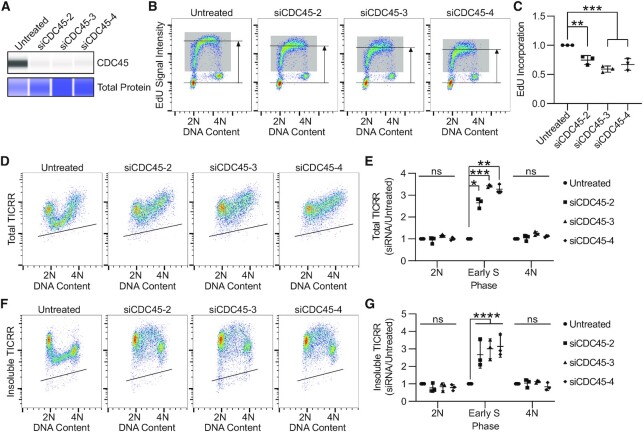
TICRR degradation requires CDC45. (**A**) Capillary electrophoresis of whole cell lysates 24 h after siCDC45 transfection probing for CDC45 (anti-CDC45) normalized to total protein. (**B**) EdU flow cytometry measurement 24 h after siCDC45 transfection. Immuno-flow cytometry of DNA content (propidium iodide) and TICRR-mClover (anti-GFP) 24 h after siCDC45 transfection. Gray box represents EdU+ cells. Horizonal lines represent median intensity of G1 (bottom) and EdU+ (top) cell populations. (**C**) Quantification of the median EdU signal intensity from cells in B (*n* = 3 independent experiments). Signal was normalized to that of untreated cells. (**D**) Immuno-flow cytometry measurement of total TICRR-mClover (anti-GFP) and DNA content (propidium iodide) 24 h after siCDC45 transfection. (**E**) Quantification of median TICRR-mClover signal in 2N, early-S, and 4N cells from D (*n* = 3 independent experiments). Values are normalized to untreated cells. (**F**) Immuno-flow cytometry measurement of insoluble TICRR-mClover (anti-GFP) and DNA content (propidium iodide) 24 h after siCDC45 transfection. (**G**) Quantification of median TICRR-mClover signal in 2N, early-S, and 4N cells from F (*n* = 3 independent experiments). Values are normalized to untreated cells. Black lines within bivariate density plots in D and F represent upper limits of background signals from negative control. Midlines and error bars in scatterplots in C, E and G mark the mean ± SD. *P*-values are from two-way ANOVA with Dunnett's Post Hoc Test (^∗^*P*< 0.05, ^∗∗^*P*< 0.01, ^∗∗∗^*P*< 0.001 and ^****^P< 0.0001).

Given that PCNA knockdown inhibited TICRR degradation in S phase without substantially reducing DNA synthesis, we asked whether another DNA replication factor, CDC45, was required for TICRR degradation. CDC45 is absolutely required for DNA replication, as it is involved in the early steps of replication fork initiation and ultimately becomes an integral component of the replicative helicase. Therefore, we used siRNA to knock down CDC45 and then, after 24 h, measured effects on DNA replication via flow cytometry. CDC45 knockdown significantly reduced CDC45 protein levels as measured by capillary electrophoresis (Figure 8A), and knockdown of CDC45 inhibited DNA synthesis (Figure 8B, C). Next, we evaluated how knockdown of CDC45 influenced total and insoluble TICRR protein levels. After 24 h, both total and insoluble levels of TICRR increased during S phase with minimal effects on protein levels in 2N and 4N cells (Figure 8D–G). Thus, this early step of CDC45 recruitment in DNA replication initiation is required for TICRR protein degradation.

### CDK1/2 inhibition promotes TICRR protein degradation during S phase

**Figure 9.**
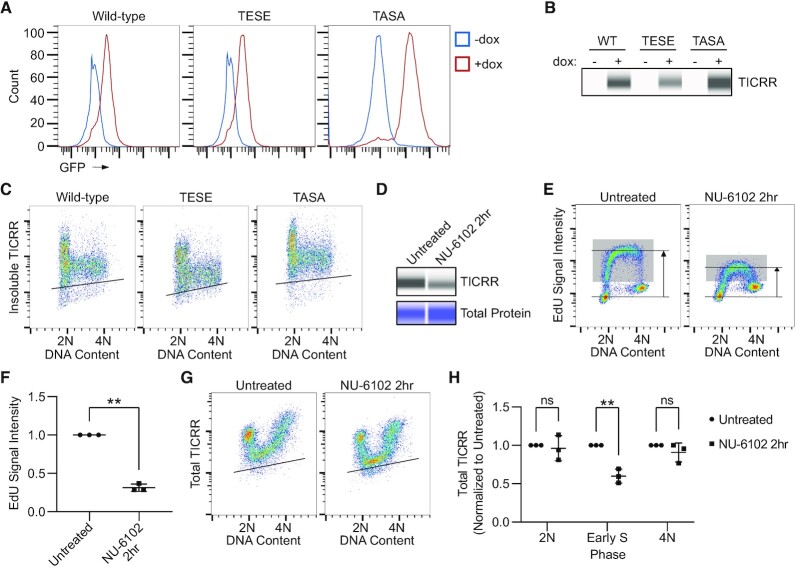
CDK1/2 inhibition promotes TICRR degradation in S phase. (**A**) Counts of live intact 293 Flp-In T-Rex cells expressing mfGFP fused to wild-type TICRR (WT), phosophomimetic TICRR (TESE: T969E, S1001E), or phosphomutant TICRR (TASA: T929A, S1001A) +/– doxycycline induction with indicated GFP fluorescence intensities measured by flow cytometry. (**B**) Capillary electrophoresis of whole-cell lysates after 24 h doxycycline induction probing for mfGFP-TICRR (anti-GFP). (**C**) Immuno-flow cytometry measurement of DNA content (propidium iodide) and insoluble mfGFP-TICRR (anti-GFP) in 293 Flp-In T-Rex cells after 24-h doxycycline induction. (**D**) Capillary electrophoresis using an anti-GFP antibody of TICRR-mClover HCT-116 whole-cell lysates after NU-6102 (20uM) treatment for 2 h. (**E**) EdU flow cytometry after 2 h of NU-6102 (20uM) treatment. The gray box represents EdU + cells. Horizontal lines represent the median intensity of G1 (bottom) and EdU+ (top) cell populations. (**F**) Quantification of the median EdU signal intensity from cells in E (*n* = 3 independent experiments). The Signal was normalized to that of untreated cells. Two-tailed paired Student's *t*-test, **P< 0.01. (**G**) Immuno-flow cytometry of DNA content (propidium iodide) and TICRR-mClover (anti-GFP) after 2 h of NU-6102 (20uM) treatment. (**H**) Quantification of median TICRR-mClover signal in 2N, early-S, and 4N cells from G (*n* = 3 independent experiments). Values are normalized to untreated cells. *P*-values are from two-way ANOVA with Dunnett's Post Hoc Test (**P< 0.01). Black lines within bivariate density plots C and G represent the upper limit of the background signal from the negative control. Midlines and error bars in scatterplots in F and H mark the mean ± SD.

S phase CDK is required for activation of the replicative helicase, and our experiments showed that CDK1/2 inhibition by NU-6102 during G1 prevented the onset of DNA replication and TICRR degradation (Figure 1I). Paradoxically, Charrasse *et al.* showed that CDK protects TICRR from proteasomal degradation, and a TICRR mutant with phosphomimetic substitutions at CDK sites (T969E; S1001E) was resistant to degradation (49). Hence, it is unclear whether CDK inhibits or promotes TICRR degradation during S phase. To address this question, we examined the cell cycle expression pattern of the TICRR proteins with mutations in the two key CDK phosphorylation sites (T969 and S1001) (49). Using flow cytometry as in Figure 1F-G, we measured the levels of insoluble mfGFP-tagged wild-type, phospho-mimetic (T969E; S1001E), or phospho-dead (T969A; S1001A) TICRR expressed from doxycycline-inducible transgenes in 293 Flp-In cells. All three proteins showed the same pattern of expression; their levels were highest during G1 and then dropped sharply at the G1/S transition (Figure 9A–C). Therefore, our data suggest that CDK phosphorylation of TICRR at T969 or S1001 is insufficient to inhibit TICRR degradation by CRL4^DTL^ during S phase.

Our previous experiments only addressed whether CDK activity during G1 was required for TICRR degradation. To determine whether CDK inhibition affected TICRR levels during S phase, we treated asynchronously cycling HCT-116 TICRR-mClover cells with NU-6102 for 2 h and measured TICRR protein levels by capillary electrophoresis (Figure 9D). Two-hour treatment with NU-6102 resulted in a decrease in bulk TICRR protein levels (Figure 9D). Like CDC45 knockdown, we expected CDK1/2 inhibition to suppress DNA replication initiation. Indeed, pulse-labeling these cells with EdU for 15 min demonstrates that CDK1/2 inhibition inhibits DNA synthesis even more than CDC45 knockdown (Figure 9E, F). Next, we measured total TICRR protein levels using immuno-flow cytometry after treatment with NU-6102 on asynchronous cells (Figure 9G, H). In contrast to the effect of NU-6102 during G1, which prevented TICRR degradation, NU-6102 treatment during S phase caused a stronger reduction in TICRR levels, supporting the hypothesis that CDK protects TICRR from degradation. Importantly, the effect of NU-6102 on TICRR levels was specific to S phase, as it had no effect on TICRR expression in 2N or 4N cells (Figure 9G, H). Overall, these data show that CDK protects TICRR from degradation, specifically during S phase.

## DISCUSSION

In the work described here, we applied endogenous gene tagging and flow cytometry to measure changes in TICRR and MTBP protein levels throughout the cell cycle. Unexpectedly, we found that TICRR levels rapidly decrease as cells enter S phase. In contrast, the levels of MTBP change little at the G1/S transition. The time at which TICRR levels drop is highly correlated with the onset of DNA synthesis. Furthermore, blocking S phase entry with CDK inhibitors or inhibiting DNA replication initiation by siRNA knockdown of CDC45 prevented TICRR degradation. Altogether, our work suggests that TICRR degradation is coupled with DNA replication initiation.

Our work and the published work of Charrasse *et al.* have shown that overall TICRR levels are increased in cells treated with proteasome and neddylation inhibitors, suggesting that a cullin E3 ubiquitin ligase is required for TICRR degradation (49). We have shown that the effects of both inhibitors were cell-cycle-phase specific, as they first increased TICRR levels during early S phase. This indicates that S phase levels of TICRR are determined by its degradation during S phase. Through two targeted siRNA screens, we identified the CRL4^DTL^ E3 ubiquitin ligase complex as a regulator of TICRR protein degradation during S phase. Most CRL4^DTL^ substrates interact with PCNA, and their interaction with DNA-bound PCNA is necessary for their ubiquitination (61). We showed that PCNA knockdown or PCNA-PIP inhibitor treatment suppresses TICRR destruction. Altogether, our data support a model in which TICRR degradation during S phase is triggered by the recruitment of a substrate to CRL4^DTL^ by a PCNA-PIP interaction. This mechanism may be similar to those causing the destruction of other CRL4^DTL^-PCNA substrates such as p21, SET8, or CDT1. Unlike those substrates, TICRR lacks an obvious PIP degron. Although we have not ruled out that TICRR destruction by CRL4^DTL^ is triggered by ubiquitination of another substrate, we have shown downregulation of neither p21 nor SET8 nor CDT1 is sufficient to degrade TICRR. Therefore, TICRR destruction likely involves another CRL4^DTL^ substrate or a non-canonical PIP degron within TICRR itself.

In addition to measuring total TICRR and MTBP levels, we used flow cytometry to assess the amount of each protein in the ‘detergent-resistant’ or insoluble fraction of the nucleus. Using a buffer frequently used to extract all except chromatin- or nuclear matrix-associated proteins, we showed that both TICRR and MTBP were resistant to extraction during G1. Recently, MTBP was shown to bind to DNA replication initiation sites in human cells, and it was proposed that the TICRR-MTBP complex is recruited to origins through a DNA-binding sequence in the MTBP C-terminus (64). The yeast homolog of TICRR, Sld3, is known to bind to early-firing replication origins during G1, so it is possible that the association of TICRR-MTBP with the G1 detergent-resistant fraction represents their binding to origins (22). Like TICRR, the level of MTBP in the detergent-insoluble nuclear fraction decreased at the G1/S transition, and that decrease depended on the CRL4^DTL^ complex. However, unlike TICRR, total MTBP levels did not fall at the G1/S transition. Although our data do not reveal a causal relationship between TICRR levels and MTBP binding to chromatin, it does suggest that MTBP on chromatin at the onset of S phase may be reduced at least in part because CRL4^DTL^ destroys its binding partner, TICRR. Alternatively, TICRR degradation could be triggered by its disassociation from MTBP. We do not yet know whether other initiation factors are released from chromatin through a CRL4^DTL^-dependent mechanism.

Interestingly, our data show that although CDK is necessary for replication initiation, it inhibits rather than promotes TICRR destruction. We are not the first to show that CDK stabilizes TICRR. Charrasse *et al.* showed that knockdown of ENSA, a PP2A inhibitor, caused cullin-dependent ubiquitination and proteasomal degradation of TICRR. Since CDK phosphorylates TICRR, and PP2A dephosphorylates CDK substrates, Charrasse *et al.* hypothesized that PP2A destabilizes TICRR by dephosphorylating it. In support of their hypothesis, they showed that TICRR with phosphomimetic mutations in two CDK sites was resistant to proteasomal degradation. Although the same phosphomimetic mutant was efficiently degraded at the G1/S transition in our experiments, we showed that overall CDK1/2 inhibition during S phase promoted TICRR destruction. Therefore, we propose that CDK suppresses TICRR degradation by phosphorylating other sites in the protein or even other substrates. The mechanism by which CDK suppresses CRL4^DTL^-induced degradation of TICRR is still unknown.

TICRR is required for DNA replication fork initiation throughout S phase, so why would it be degraded when it is needed? Although its levels are low, TICRR is expressed during S phase (Figure 1D). An implication of TICRR destruction during S phase is that the protein may not be recycled as proposed in current ‘limiting factor’ models of replication programs (21). Instead, TICRR protein levels may be actively regulated during S phase. We propose that the CRL4^DTL^ ubiquitin ligase complex induces TICRR degradation in response to DNA synthesis, thereby limiting the amount of TICRR available for origin firing. This model is consistent with published studies showing that TICRR/Sld3 is one of a few limiting initiation factors (20–22). Notably, suppressing TICRR destruction by CRL4^DTL^ inhibition does not stimulate DNA replication (Figure 4F, G). This could be due to inhibitory effects of CRL4^DTL^ knockdown on DNA replication, such as p21 overexpression or re-replication-induced checkpoint activation (65–67). Alternatively, TICRR overexpression by itself may be insufficient to promote origin firing. To our knowledge, it has not been demonstrated that Sld3 or TICRR alone deregulates replication initiation. Furthermore, the CRL4^DTL^-TICRR negative feedback loop we propose would not be the only regulatory mechanism maintaining replication fork homeostasis. DNA replication activates the ATR and CHK1 kinases, and ATR or CHK1 inhibitors increase DNA replication origin firing, primarily by increasing CDK activity (68–71). Therefore, we hypothesize that CRL4^DTL^-TICRR and ATR-CHK1-CDK negative feedback loops work in parallel to regulate the amount of CDK-phosphorylated TICRR. In agreement with this model, our previously published work showed that in U2OS cells, overexpression of wild-type TICRR is insufficient to stimulate DNA replication initiation, yet overexpression of TICRR with phosphomimetic mutations in two CDK sites increased origin firing rates (33). Future work should focus on understanding the individual or combined effects of ATR-CHK1-CDK, CRL4^DTL^-TICRR, and the regulation of other limiting initiation factors on the spatiotemporal regulation of origin firing.

## DATA AVAILABILITY

Flow cytometry data is deposited in Flow Repository, experiment codes: FR-FCM-Z477, FR-FCM-Z478, FR-FCM-Z479 and FR-FCM-Z47A.

## Supplementary Material

gkab805_Supplemental_File
