# Supplementary material for: The CRL4DTL E3 ligase induces degradation of the DNA replication initiation factor TICRR/TRESLIN specifically during S phase
Source: Nucleic Acids Res. 2021 Sep 17;49(18):10507–23. doi: 10.1093/nar/gkab805 (PMC8501952; doi:10.1093/nar/gkab805)
Supplement: gkab805_Supplemental_File [file gkab805_supplemental_file.pdf]

Supplementary Tables

Supplementary Table 1. Plasmids used in this study

Plasmid Name	Description
pX330-U6-Chimeric_BB-CBh-hSpCas9	Cas9 gRNA expression plasmid. Used to generate pX330-CS1997 and pX330-CS1967. (Addgene #42230)
pX330-CS2190	Cas9 gRNA expression plasmid. Targets C-term of hMTBP for degron knock-in. Constructed for this study.
pX330-CS1967	Cas9 gRNA expression plasmid. Targets C-term of hTicrr for degron knock-in. Constructed for this study.
pBSKII+	Plasmid backbone for donor constructs. Purchased from Stratagene.
pMK289	Plasmid containing the mAID-mClover-Neo cassette used to make knock-in donor construct. (Addgene #72827)
pMK290	Plasmid containing the mAID-mClover-Hygro cassette used to make knock-in donor construct. (Addgene #72828)
pOG44	Flp recombinase expression plasmid. Purchased from Invitrogen.
pcDNA5/FRT/TO	Plasmid backbone for generating Flp-In gene constructs. Purchased from Invitrogen.

Supplementary Table 2. Synthetic DNA used in this study

Name	Description	Sequence
CS2190	Oligos for inserting gRNA sequence into pX330. Used for targeting the C-terminus of MTBP	Oligo 1: 5’-CACCgTCTCCCCCTAGGTGATTGAC-3’<br>Oligo 2: 5’-AAACGTCAATCACCTAGGGGGAGAc-3’
CS1967	Oligos for inserting gRNA sequence into pX330. Used for targeting the C-terminus of TICRR	Oligo 1: 5’-CACCGGGCTATAAGTCCTCCAGCC-3’<br>Oligo 2: 5’-AAACGGCTGGAGGACTTATAGCCC-3’
csDNA93	DNA fragment for constructing the TICRR targeting donor plasmid. Contains homology arm sequence.	AGTGGATCCCCCGGGCTGCAGGAATTCGATTGTAACCC<br>TCTGCAATTTAAGCCAATTTTTTTCAGTTCGGTCCTCTG<br>TGACTCCTGCATGCTAAGCCTTTCTACCTTCTTCTAGAT<br>GAGGATGTGGATGTTCTTCCCTCCACTGTAGAAGACTC<br>TCCTTTCAGTCGCGCTTTCTCCAGGAGGCGCCCCATCA<br>GCAGAACTTATACACGGAAGAAGCTCATGGGAACCTGG<br>CTGGAGGACTTAGGATCCGGTGCAGGCGCCAAGGAGA<br>AGAGTG
csDNA94	DNA fragment for constructing the mAID-mClover-Hygro TICRR targeting donor plasmid. Contains homology arm sequence.	GTTATTAGGTCCCTCGAAGAGGTTCACTAGTAGCCACA<br>AACATTACTGAGCCCAAAAGATCAAGGAGTCAGCCAGG<br>ACCCTGTGGACATAAAGAAGTTGGATGCCTGGTCCCAA<br>GCCTCTTTTGCCATGGTCAGTGTTCAGATTGCCATTAGA<br>ATGCCTTAGGGTTTTCTAATTCCCCTTATGGATCCAATC<br>CATCTCCTGGCCCTGCCCCTTGTTGGGGAAGTTGCAGG<br>AGGATCAAGCTTATCGATACCGTCGACCTCGAG
csDNA95	DNA fragment for constructing the mAID-mClover-Neo TICRR targeting donor plasmid. Contains homology arm sequence.	CAGAATAAAACGCACGGTGTTGGGTCGTTGTAGCCACA<br>AACATTACTGAGCCCAAAAGATCAAGGAGTCAGCCAGG<br>ACCCTGTGGACATAAAGAAGTTGGATGCCTGGTCCCAA<br>GCCTCTTTTGCCATGGTCAGTGTTCAGATTGCCATTAGA<br>ATGCCTTAGGGTTTTCTAATTCCCCTTATGGATCCAATC<br>CATCTCCTGGCCCTGCCCCTTGTTGGGGAAGTTGCAGG<br>AGGATCAAGCTTATCGATACCGTCGACCTCGAG

Supplementary Table 2. Synthetic DNA used in this study (continued)

Name	Description	Sequence
csDNA126	DNA fragment for constructing the MTBP targeting donor plasmid.	AGTGGATCCCCCGGGCTGCAGGAATTCGATGTACTCAGTATTGGAAGTCCAACAAAAATGTTCTTCCATTAGAAAATAAAATGTGTATTTAAGTATAAGATTTCAGTATCTTCCTTTTTATTTGAAATTTTCTACCATGTTCTTTTGAAGTTTTTACAATATATTTTTCTATTACTTGTGTTTTCAAGAGAAATCTTCTGTAATCATATTTTTTCTCCCCCTAGGTAATCGATTGGGTGTTGGAGAAAACCAGTAAAAAGGGATCCGGTGCAGGCGCCAAGGAGAAGAGT
csDNA127	DNA fragment for constructing the MTBP targeting donor plasmid.	GTTATTAGGTCCCTCGAAGAGGTTCACTAGACTGGGTATTAGAAAAGACAAGCAAGAAATGATACATAATCATTCTCTTTAAGACAATTATAAATTGGATGGAGCTATTATTCACTACTTCTTTTCTTAGTTTGAAAATTATAAACAAATTTTAAGCTTTATTCAAAGAATAGATGTTATATTTCTAAAGAATTTCATGAATATATATTCTATATTTGTATAGTTTGAGGATCAAGCTTATCGATACCGTCGACCTCGAG
mAID-mClover-Neo cassette Primers	PCR primers for amplifying the knock-in cassette from pMK289.	Oligo 1: 5’-GGATCCGGTGCAGGCGCC-3’<br>Oligo 2: 5’-CAACGACCCAACACCGTGCG-3’
mAID-mClover-Hygro cassette Primers	PCR primers for amplifying the knock-in cassette from pMK290.	Oligo 1: 5’-GGATCCGGTGCAGGCGCC-3’<br>Oligo 2: 5’-CTAGTGAACCTCTTCGAGGGACCTA-3’

Supplementary Table 3. siRNA used in this study

siRNA	siRNA ID	Sequence
CUL1	SASI_Hs02_00335921<br>SASI_Hs01_00021744	GAGAAGAUUUGAUGGAUGA<br>GAAUUGAAGCCAGAUACCU
CUL2	SASI_Hs01_00093148<br>SASI_Hs01_00093149	CUGAAGAAGCCAUGAUCAA<br>GAUAUUGAUGCAGAAUCUU
CUL3	SASI_Hs01_00102205<br>SASI_Hs01_00102207	GAGUGUAUGAGUUCCUAUU<br>GAAUAACAGUGGUCUUAGU
CUL4A	SASI_Hs01_00146692<br>SASI_Hs01_00146693	CAAAGCACGUGUGCUGAUU<br>CCUAUAGACCUCACAGUGA
CUL4B	SASI_Hs02_00314020<br>SASI_Hs02_00314021	CAAAUGAAGCAGUGUAAUU<br>GGAUUCAUUGGAUAGCGUU
CUL5	SASI_Hs01_00112044<br>SASI_Hs01_00112046	GACUUUAUGGUCUUUAGUA<br>GACAAAGUUCCUAAUGGUA
CUL7	SASI_Hs01_00152019<br>SASI_Hs01_00152020	GACUUUGUGCCACGCUACU<br>CAAUACCUAUGCUUUGUAU
DDB1	SASI_Hs01_00242315<br>SASI_Hs01_00242316	GUUCCUAUAUGGUUGCCAA<br>CAGACUUUCUUCUGUGGCA
SKP1	SASI_Hs01_00239156<br>SASI_Hs01_00239157	CUGUAUCAAGUAGUCUUGA<br>GUGUCUUAAACAUACUCUU
ELOB	SASI_Hs01_00184724<br>SASI_Hs01_00184725	CCUUUGAGGCCCUGUGCAU<br>GGGACAAGAGAUUCCAGUU
ELOC	SASI_Hs01_00205654<br>SASI_Hs01_00205645	CAUCUGAUGGCCAUGAAUU<br>CCUGUAGUUCAGUUAGUAA

Supplementary Table 3. siRNA used in this study (continued)

siRNA	siRNA ID	Sequence
NEDD8	SASI_Hs01_00117478<br>SASI_Hs01_00117479	CUCUUUACCCUGUCGCUCA<br>GUGGCAAGCAGAUGAAUGA
BRWD1	L-010963-00	GCGCAUCGGUCCUAUGUUG<br>CCCAGUAUUUCAAGAGUCA<br>UCAUCGAGUCGGAGCUGUA<br>GCUACGAGGAGUUGGUCUU
BTRC	L-003463-00	UGACAACACUAUCAGAUUA<br>CACAUAAACUCGUAUCUUA<br>GACCUUAAAUGGACACAAA<br>ACACCGAGCUGCUGUCAAU
CRBN	L-021086-00	CAAUUAGAAUCCCUCAAUA<br>GUAUAAGGCUUGCAACUUG<br>GACAUUACCUCUUCAGCUU<br>CGACUUCGCUGUGAAUUAG
DCAF1	L-021119-01	GGAGGGAAUUGUCGAGAAU<br>CCACAGAAUUUGUUGCGCA<br>GGAAUGACACUGUGCGCUU<br>CGGAGUUGGAGGAGGACGA
DCAF4	L-013016-01	AGGACCACGAAGUGCGUAA<br>GUUAUUAUCCAAAGGCGUU<br>UCAAGGUGUUCAUGCACGA<br>UUGUAAUCUAGGAGCGACA
DCAF5	L-021971-01	CCACAACAAUGGACGCUUA<br>CUCAAAGGACAAUCGGAUA<br>CAUAGAAUCAGUUGAGCGA<br>CUGAAAGGGCAUCGAUCUA
DCAF6	L-020878-01	CGUUCAGGGCACCGAGCAA<br>GAAUAUAUGAUCGGCGAAU<br>GUGCAUGAUGGUUGUGUUA<br>AUACCAACGUUGAGCAAGA
DCAF7	L-019999-00	GGAAUGAGGUGGAUCCUUA<br>GCUGAUCGCCCAUGACAAA<br>GGACAGUCUACGCGAUGAA<br>UUAGGUACCUCAAGCAUUG
DCAF8	L-013062-01	GUGCAUAGGACUCGGCAGA<br>GUACAGCGCAAGCGGGCUA<br>GGGAAUGUGUGGUGAUCGA<br>GCAGAACAGACUUAGCUAA
DCAF10	L-014344-01	AAAUAGUCUUGAAGUCGUA<br>GGGUGAAGAACAUCGAAUA<br>CAGAAUGAGCUUUGGUUAU<br>UCUAUAUUGAGCACAGAUA
DCAF11	L-019051-01	GCAAAGUGGUUGUGUACGA<br>GCAAGUAGAUAUCGAGAGA<br>CCAUAUAGGGUGAACCUCU<br>UGCAAUAGGUGCUCCCUUA
DCAF13	L-017898-01	CGAUAAAUCUAUUCGAAUC<br>GGAAUCUAACUCAGCGGAA<br>CGAAAGGAAGUGAAUCGUA<br>ACGUAUAGCUCGUCAUCGA
DCAF15	L-031237-01	UGGCGGACAGCGAGCGAUA<br>UCACACUAGACUUCGAAUA<br>CCUCCAAGGUCAUCGUCUU<br>AUGAGUUGGAGGACGACAA

Supplementary Table 3. siRNA used in this study (continued)

siRNA	siRNA ID	Sequence
DCAF16	L-020725-02	GAGUAUAUGCCCUUGUAAA<br>GUAGAAAUCAGUAUACGUA<br>UCUAAAUGGAGCACUGCAA<br>GUCCAUAUACUUCGAGAGA
DCAF17	L-014543-02	ACACAUAGCGAGAGACAAU<br>GUGGAAUACUUCUGGUCUA<br>GUACAGACGUUACGCUGAA<br>ACUUAUGGAACAAGCAUUA
DDA1	L-014277-01	CCUCAUAGGAGCCGAUGUA<br>CAAACAUCCUCCUGCGCUA<br>GCGAGUACCCGUCUGAACA<br>CUGUAUCAAGCGUUGGUUA
DDB2	L-011022-00	GAUAUCAUGCUCUGGAAUU<br>GCCGAUACCCAGAUCCUAA<br>GAAGACCUCCGAGAUUGUA<br>GGCAUCAGUUCGCUUAAUG
DET1	L-019393-00	GUAGUAACACUGCGAGUCA<br>GAUAAUGGAUCAUCAUGUU<br>CAAGUACACUAGUGAGGAU<br>GAACAGGAUGGUAGUGCAA
DTL	L-020543-00	GCGCUUGAAUAGAGGCUUA<br>ACUCCUACGUUCUCUAUUA<br>GAAUUAUACUGCUUAUCGA<br>GUCAAGACCUGGCCUAGUA
EED	L-017581-00	GAUCAUGCUUUACGAUUAU<br>CAACAGAGUUACCUUGUAU<br>GGAUCUAGAGGCAUAAUUA<br>GAGACAUACAUAGGAAUUA
ERCC8	L-011008-00	GUAAAGCAGUGUGUUCCAU<br>CAGACAAUCUUAUUACACA<br>CAUCAUAUGUCUCCAGUCU<br>GAUUGUACUUUAUGACCUU
GRWD1	L-027146-01	CACCAAAGAACUCGGUUUA<br>GGAUUCUGUUUGACGUAUU<br>GCUUUGACAUAGUCCGGGA<br>UGUCAUGGCUGGGUGAAGA
NLE1	L-015863-00	GACAAAUGCCUCCGGAUAU<br>CGGAUGAGGUAUAUGCUGU<br>ACACCAAGCUCUCAUCAAC<br>CCGACGACUUCACCUUAUU
NUP43	L-018906-01	GGAGGAAAUUUAUGCGAAG<br>UGGAGAGGAUGGUCGAAUA<br>AUGUAAUGUAACACCGUAA<br>CAAGAUGGAAUGUUGAGUA
PHIP	L-019291-00	CAACACAAUUAUCGUACAA<br>UAAACUGACUGGCGGAUCA<br>GCACGUAUUUGGCAAUUUA<br>GAUGGGAGGUUGUUAGCUA
PWP1	L-019744-01	CAGUACAGAUGACGGCUUU<br>GCAUUUGGAAGACGAGAGA<br>ACUGUAAUUCUGUGGGAUA<br>CUACGGGAGUAAUGAUCAA
RBBP4	L-012137-00	GAUACUCGUUCAAACAAUA<br>GCAUACGGCAGUAGUAGAA<br>GCUAUGGGCUUUCUUGGAA<br>GACUGAAUGUCUGGGAUUU

Supplementary Table 3. siRNA used in this study (continued)

siRNA	siRNA ID	Sequence
RBBP5	L-012008-00	UAACACGGCAGAUCGAAUA<br>UAUAGAACUUCAAGGAGUA<br>GCAAUACCACAGCCAUUAA<br>GAUGGAACUUUGGAUUGUA
RBBP7	L-011375-00	CCACUGGUCUCCACAUAAU<br>CGGAUAAGACCGUAGCUUU<br>GAAGUAAACCGUGCUCGUU<br>AUACACCGUUUCUAUAUGA
RFWD2	L-007049-00	CUACAAGGAUGUCUCGUAU<br>GCUAAUGUGUGCUGUGUUA<br>GAAUUGGUAUGAAGGGUUA<br>CAUAAGAACCUGUUAGCUA
SMU1	L-021129-01	GAACUUUGACAGUGCACGA<br>GGACAGAUAUUACCGUCAA<br>CAGUUUACAUCGGGCGUUA<br>AAACACAGCCAGAGCGAUA
SNRNP40	L-019860-00	GAGAGGCCAUGCAGAUUCA<br>CCAAUGCAAUGGACAAUAC<br>CGACAGGUUUGUUUAUGUG<br>GAUUUGACCGACUGAUAUU
TLE1	L-015528-00	GUAAAUGGUUUGUGAGUAC<br>GCACGCCUGUUCUGAAAUC<br>GAUAAGGACUCCAGCCACU<br>GUAAAUUGCUACCCGAUGG
TLE2	L-015873-00	GCAUUGAUAUUUCCGAUUA<br>CGAGACAACUACAUUCGUU<br>CCAUCAGGACCUUAUGAAA<br>GUGAAGCGUCUGAGCGGUA
TLE3	L-019929-00	GCCAUUAUGUGAUGUACUA<br>GCAUGGACCCGAUAGGUAU<br>GAACCACCAUGAACUCGAU<br>UCAGGUCGAUGCCGGGUAA
WDR12	L-012972-00	GAUCUUGACUGGUUCUUAU<br>GACCAUACAAUUAGAGUGU<br>AAACACGCUUCUACACUGA<br>GUUAAGCUGUGGGAUACAA
WDR26	L-032006-01	AGAGUAGGAAUCAGCGAUA<br>GCAGAAGACCUACGUGCAA<br>UGAUUAAGGGCUAGAGCAA<br>GGUAAAGAAGCCUAGGAUU
WDR5	L-013383-00	GACGAAAGCGUGAGGAUAU<br>GUGGAAGAGUGACUGCUAA<br>GACGUGAGCUCGGGCAAGU<br>GAUGGAUCCUUGAUAGUUU
WDR5B	L-013375-00	CAUCGCAUCAGCAGCAUUA<br>AAACAUACACUGGUCAUAA<br>CAAACUAUGCUCUCAAAUG<br>GGACAACACUCUUAAACUA
WDR53	L-018933-01	GGUGGAAAUACUAACGCUU<br>GGUGAUGGGAGUUAAGUGU<br>GUGAACUGGCUCUUGGGUA<br>UGGUAAGGUUCGAAUCUUU
WDR61	L-014614-01	AGGAACUCAUGUCGGGAAA<br>CAAAGAGAAUGUACGGAUU<br>CGUCUUUGGGACUUGGAAA<br>GAUCAGGUCUGGGGAGUAA

Supplementary Table 3. siRNA used in this study (continued)

siRNA	siRNA ID	Sequence
WDR76	L-014509-00	GAGCUAUGAUGGCACGUUA<br>CGACAUUAGUAGCAGAUGA<br>GGGAUUGGAUGUAGAAGGU<br>GAUGCAAGGCGAUUGAAUU
WDR82	L-016629-01	UGAAAGUGCAAGAGGCGUA<br>GUGCAUACAGAUACGCAUA<br>CCUCGUUGCCAAUGAUAGA<br>CCCAAAUGUAGACUAGAAU
WDTC1	L-016542-01	CAGCCUACAUGAAGCGCAA<br>GAGUGAGGGUGACGCAAUU<br>CACCAUACCUGGAGCGUGU<br>CAAACCGGGUGAAGCGCAU
SET8	-	ACUUCAUGGCGCUCCGUAC<br>GAUUUGUCUCUCUAGUUGC
p21	-	CUAAGAGUGCUGGGCAUUU<br>GCGAUGGAACUUCGACUUU
CDT1	-	GCAAUGUUGGCCAGAUCAA<br>CCUACGUCAAGCUGGACAA
CDC45	-	GUCAAUGUAUACAACGAUAUU<br>CGUCAGCCAUGGUGAUGUU<br>GCAAAGAGUUCUACGAGGU
PCNA	-	CGGUGACACUCAGUAUGUC<br>GGAGGAAGCUGUUACCAUA

Supplementary Table 4. Figure 7D Statistics

2N Insoluble Protein:

Dunnett's multiple comparisons test	Summary	Adjusted P Value
**TICRR**		
Untreated vs. siCUL4A-1 + siCUL4B-1	ns	0.9968
Untreated vs. siCUL4A-2 + siCUL4B-2	ns	0.2581
Untreated vs. siDDB1-1	**	0.0080
Untreated vs. siDDB1-2	ns	0.1126
Untreated vs. siDTL	*	0.0160
**MTBP**		
Untreated vs. siCUL4A-1 + siCUL4B-1	ns	0.0597
Untreated vs. siCUL4A-2 + siCUL4B-2	***	0.0004
Untreated vs. siDDB1-1	*	0.0234
Untreated vs. siDDB1-2	**	0.0040
Untreated vs. siDTL	**	0.0019

Supplementary Table 4. Figure 7D Statistics (continued)

S-phase Insoluble Protein:

Dunnett's multiple comparisons test	Summary	Adjusted P Value
**TICRR**		
Untreated vs. siCUL4A-1 + siCUL4B-1	*	0.0291
Untreated vs. siCUL4A-2 + siCUL4B-2	**	0.0046
Untreated vs. siDDB1-1	****	<0.0001
Untreated vs. siDDB1-2	****	<0.0001
Untreated vs. siDTL	****	<0.0001
**MTBP**		
Untreated vs. siCUL4A-1 + siCUL4B-1	ns	0.5622
Untreated vs. siCUL4A-2 + siCUL4B-2	ns	0.2845
Untreated vs. siDDB1-1	*	0.0193
Untreated vs. siDDB1-2	ns	0.0559
Untreated vs. siDTL	**	0.0012

4N Insoluble Protein:

Dunnett's multiple comparisons test	Summary	Adjusted P Value
**TICRR**		
Untreated vs. siCUL4A-1 + siCUL4B-1	ns	0.5776
Untreated vs. siCUL4A-2 + siCUL4B-2	ns	0.1594
Untreated vs. siDDB1-1	****	<0.0001
Untreated vs. siDDB1-2	**	0.0031
Untreated vs. siDTL	****	<0.0001
**MTBP**		
Untreated vs. siCUL4A-1 + siCUL4B-1	ns	0.8187
Untreated vs. siCUL4A-2 + siCUL4B-2	ns	0.0639
Untreated vs. siDDB1-1	**	0.0048
Untreated vs. siDDB1-2	ns	0.0768
Untreated vs. siDTL	*	0.0115

Supplementary Figures

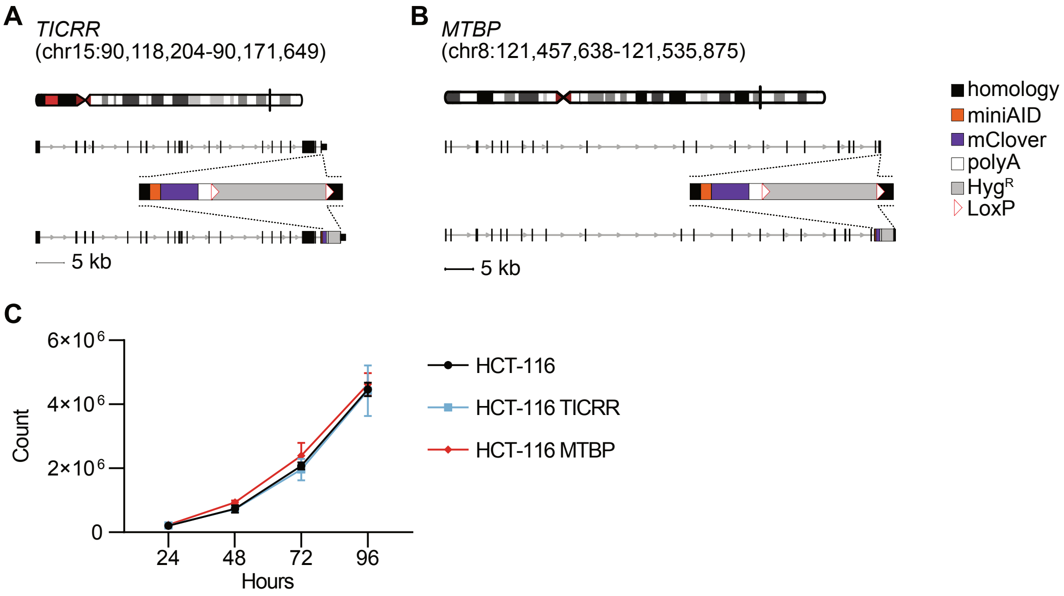

Supplementary Figure 1. Targeting constructs to generate endogenous TICRR and MTBP knock-in cell lines. Schematic of the chromosome ideogram, targeting location, and targeting constructs for (A) TICRR or (B) MTBP. Homology arms are ~200bp. (C) Three replicates of each cell line were counted at every 24 hour interval. HCT-116 TICRR-mClover and HCT-116 MTBP-mClover cell lines have no growth defects in comparison to the untagged parental HCT-116 cell line. Data represented as mean ± SD.

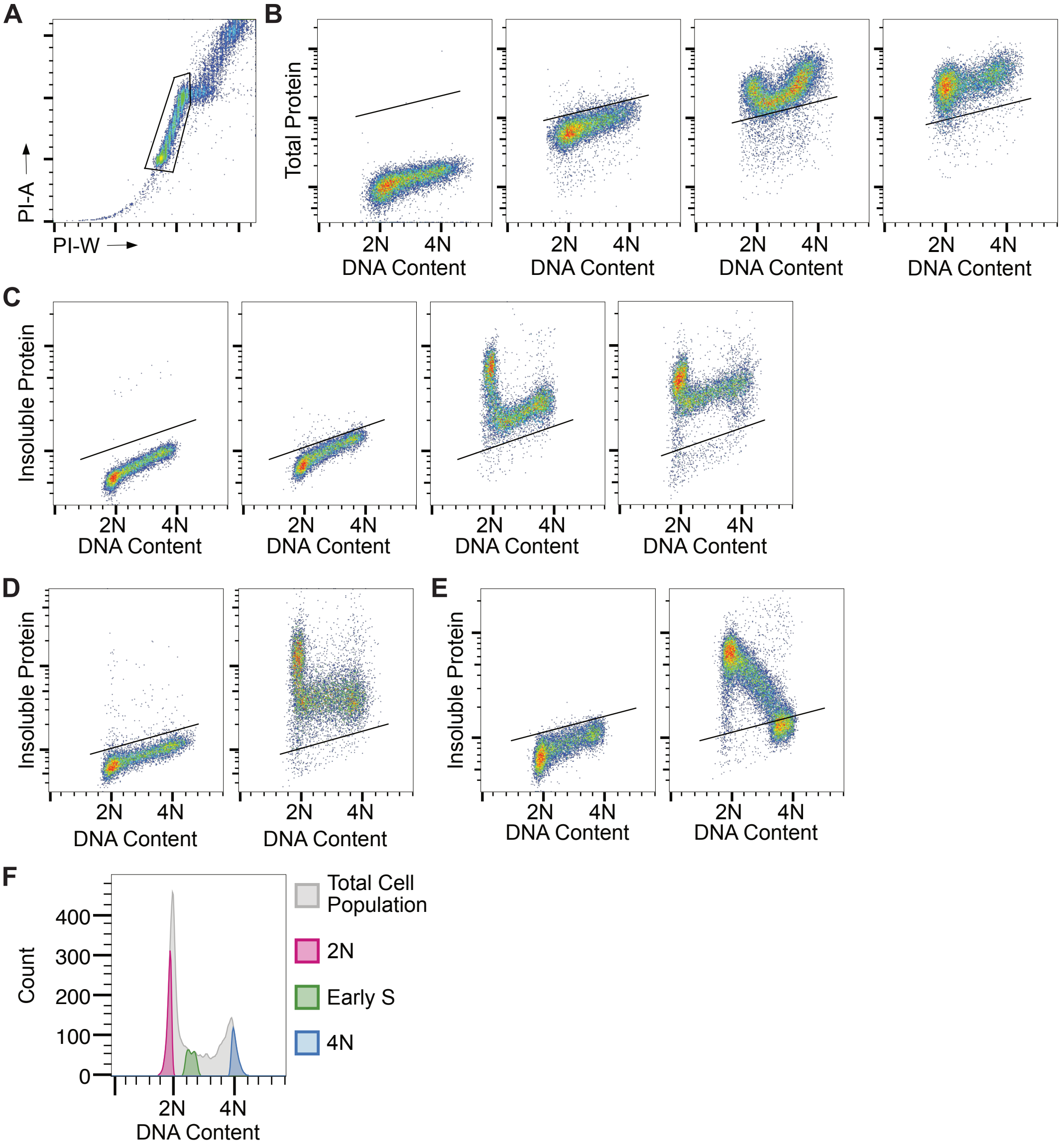

Supplementary Figure 2. Immuno-flow cytometry analysis scheme. (A) Single cells were gated based on the width (PI-W) and area (PI-A) of DNA staining with propidium iodide. (B) Gate defining positive signal for total TICRR-mClover or MTBP-mClover tagged protein. Left: secondary antibody alone in the parental HCT-116 cell line. Middle left: GFP and secondary antibodies in the parental HCT-116 cell line. Middle right: GFP and secondary antibodies in the HCT-116 TICRR-mClover-tagged cell line. Right: GFP and secondary antibodies in the HCT-116 MTBP-mClover-tagged cell line. (C) Gate defining positive signal for insoluble TICRR-mClover or MTBP-mClover tagged protein. Left: secondary antibody alone in the parental HCT-116 cell line. Middle left: GFP and secondary antibodies in the parental HCT-116 cell line. Middle right: GFP and secondary antibodies in the HCT-116 TICRR-mClover-tagged cell line. Right: GFP and secondary antibodies in the HCT-116 MTBP-mClover-tagged cell line. (D) Gate defining positive signal for mfGFP-tagged TICRR protein (example: mfGFP-TICRR(WT)). Left: GFP and secondary antibodies without doxycycline induction. Right: GFP and secondary antibodies 24 hours after doxycyline induction. (E) Gate defining positive signal for endogenous protein detection (example: MCM7). Left: secondary antibody alone in the parental HCT-116 cell line. Right: target (anti-MCM7) and secondary antibody in the HCT-116 TICRR-mClover cell line. (F) Gating 2N, early S-phase, and 4N cell populations for quantification based on propoidium iodide profiles.

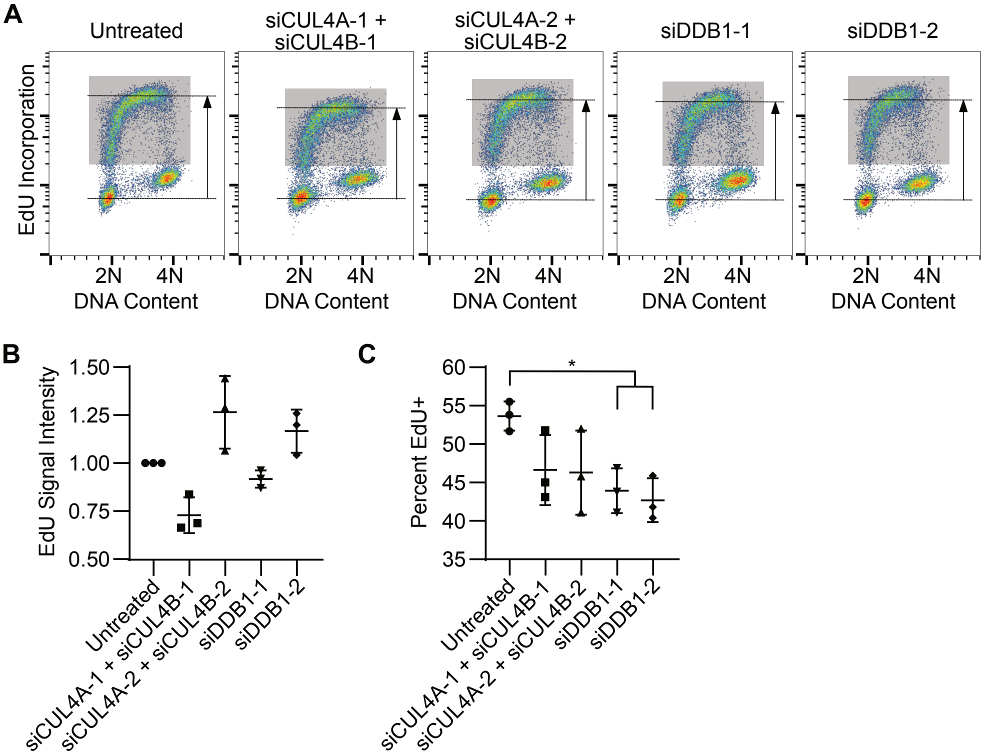

Supplementary Figure 3. DNA synthesis in CUL4A+B and DDB1 knockdown cells not significantly suppressed. (A) EdU flow cytometry 24 hours after siRNA transfection. Gray boxes represent EdU positive cells. Black lines and arrow represent median EdU signal intensities of G1 (bottom) and EdU positive (top) populations. (B) EdU signal intensity quantification from cells in A (n=3). One-way ANOVA, ns. (C) Percent of EdU positive cells from A (n=3). One-way ANOVA, * $p<0.05$.

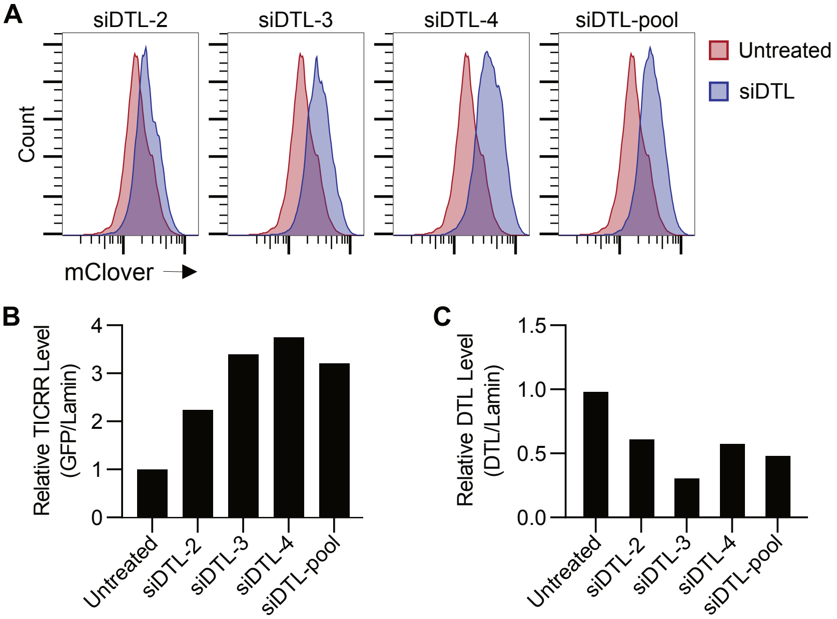

Supplementary Figure 4. Individual DTL siRNAs have similar effects as the siRNA-pool. (A) Live cell flow cytometry in TICRR-mClover cells 24 hours after DTL siRNA knockdown demonstrates individual and pool siRNAs increase mClover fluorescence. (B) Relative TICRR (anti-GFP) protein levels 24 hours after siRNA transfection as measured by capillary electrophoresis normalized to Lamin (anti-Lamin) run in the same capillary. TICRR is increased in individual and pool siRNAs. (C) Relative DTL (anti-DTL) protein levels 24 hours after siRNA transfection as measured by capillary electrophoresis normalized to Lamin (anti-Lamin) run in the same capillary. DTL levels are decreased in individual and pool siRNAs.

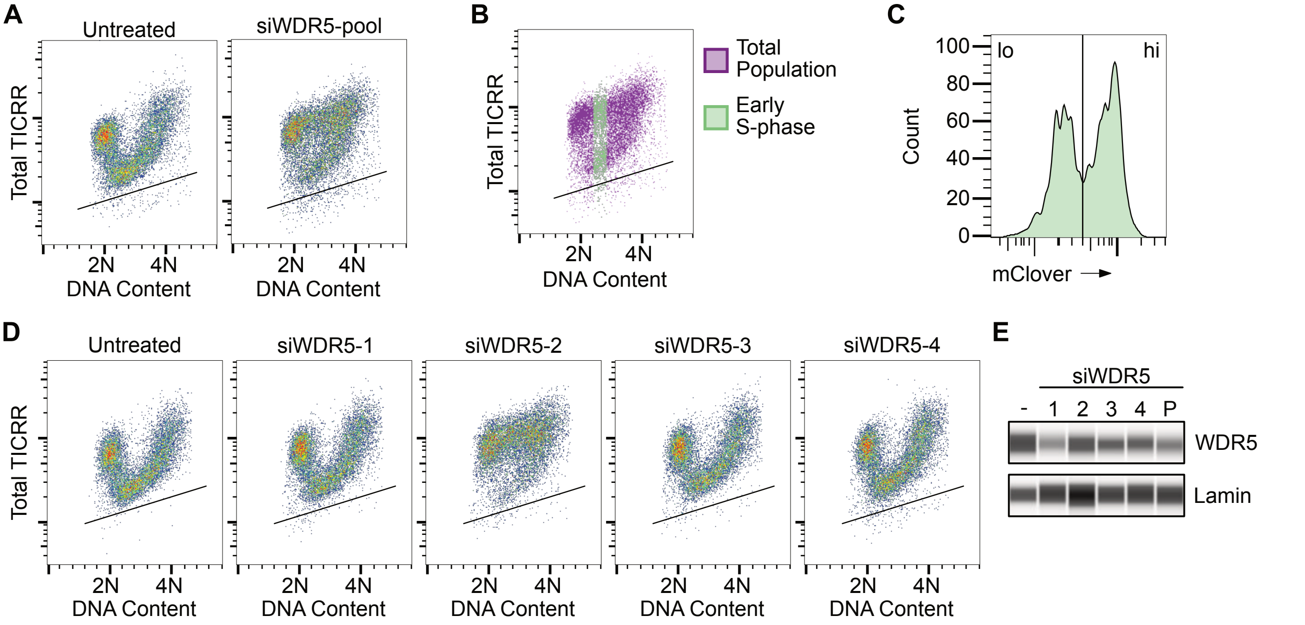

Supplementary Figure 5. WDR5 does not target TICRR for degradation during S-phase. (A) Immuno-flow cytometry measurement of DNA content (propidium iodide) and total TICRR-mClover (anti-GFP) 24 hours after WDR5 siRNA-pool transfection. (B) Immuno-flow cytometry of siWDR5 transfected cells, as in A, with early S-phase cell gate marked with green. (C) mClover histogram of early S-phase cells gated in B. (D) Immuno-flow cytometry measurement of DNA content (propidium iodide) and total TICRR-mClover (anti-GFP) 24 hours after transfection of individual WDR5 siRNAs. (E) Capillary electrophoresis of whole cell lysates from cells in D, measuring WDR5 (anti-WDR5) normalized to Lamin (anti-Lamin). P: pool. Black lines within plots A, B, and D represent upper limit of background signals from negative control.

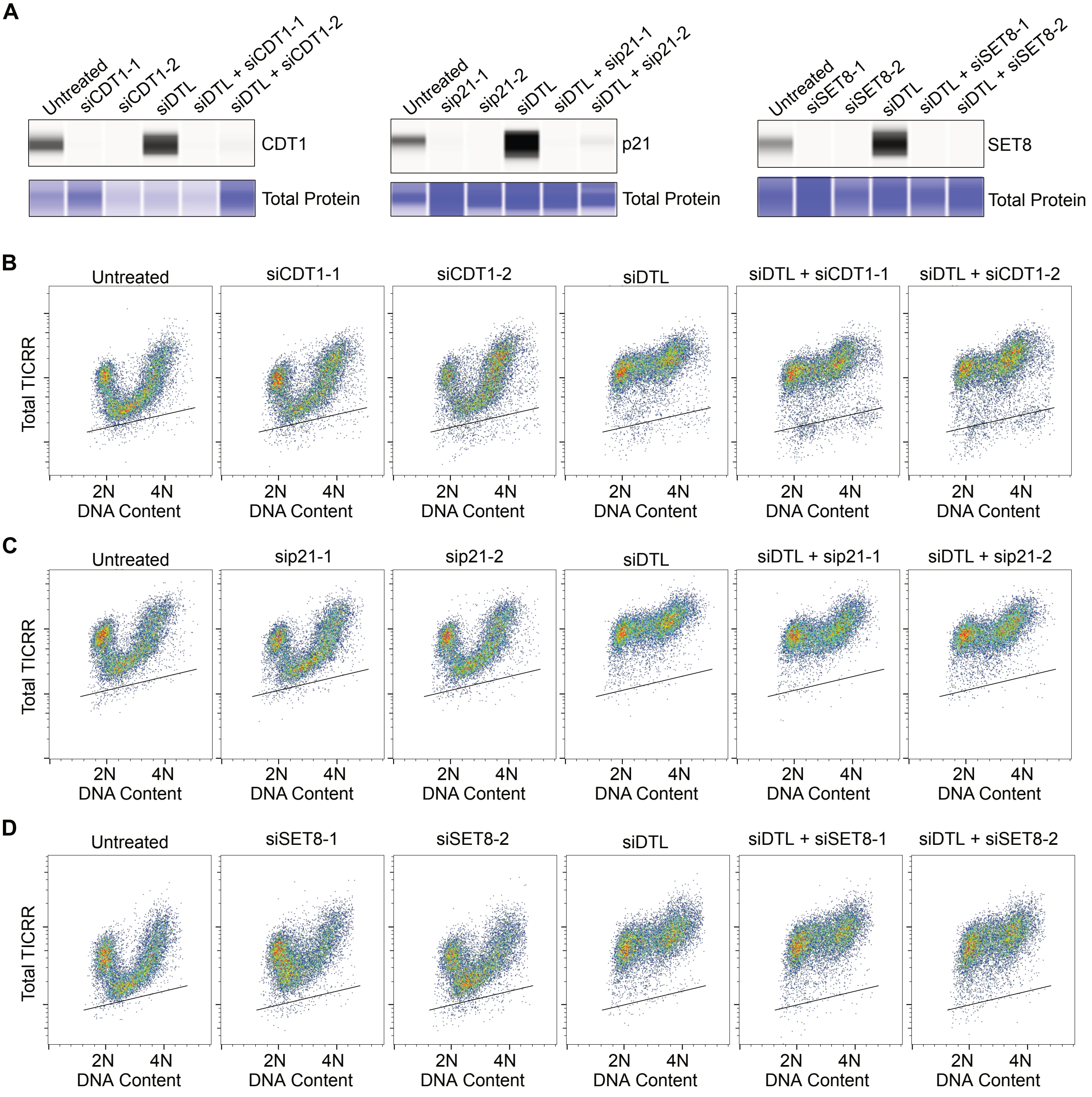

Supplementary Figure 6. TICRR is not indirectly targeted for degradation through the dysregulation of CDT1, p21, or SET8. (A) Capillary electrophoresis of whole cell protein lysates demonstrates complete knockdown of CDT1 (left), p21 (middle), or SET8 (right) with single substrate siRNA and the over-expression of each substrate with siDTL knockdown alone. Double knockdown of siDTL and siRNA against the specific substrate prevents over-expression. (B) Total TICRR flow cytometry (anti-GFP, Rockland) 24 hours after siCDT1 transfection. (C) Total TICRR flow cytometry (anti-GFP) 24 hours after sip21 transfection. (D) Total TICRR flow cytometry (anti-GFP, Rockland) 24 hours after siSET8 transfection. Black lines within plots B-D represent upper limits of background signal from negative control.
